# Two-Dimensional MXene-Based Advanced Sensors for Neuromorphic Computing Intelligent Application

**DOI:** 10.1007/s40820-025-01902-1

**Published:** 2025-09-12

**Authors:** Lin Lu, Bo Sun, Zheng Wang, Jialin Meng, Tianyu Wang

**Affiliations:** 1 School of Integrated Circuits, Shandong University, Jinan, 250100 China; 2 Suzhou Research Institute of Shandong University, Suzhou, 215123 People’s Republic of China; 3 State Key Laboratory of Crystal Materials, Shandong University, Jinan, 250100 People’s Republic of China; 4 National Integrated Circuit Innovation Center, Shanghai, 201203 People’s Republic of China

**Keywords:** Two-dimensional, MXenes, Sensor, Neuromorphic computing, Multimodal intelligent system, Wearable electronics

## Abstract

As emerging two-dimensional (2D) materials, carbides and nitrides (MXenes) could be solid solutions or organized structures made up of multi-atomic layers. With remarkable and adjustable electrical, optical, mechanical, and electrochemical characteristics, MXenes have shown great potential in brain-inspired neuromorphic computing electronics, including neuromorphic gas sensors, pressure sensors and photodetectors. This paper provides a forward-looking review of the research progress regarding MXenes in the neuromorphic sensing domain and discussed the critical challenges that need to be resolved. Key bottlenecks such as insufficient long-term stability under environmental exposure, high costs, scalability limitations in large-scale production, and mechanical mismatch in wearable integration hinder their practical deployment. Furthermore, unresolved issues like interfacial compatibility in heterostructures and energy inefficiency in neuromorphic signal conversion demand urgent attention. The review offers insights into future research directions enhance the fundamental understanding of MXene properties and promote further integration into neuromorphic computing applications through the convergence with various emerging technologies.

## Highlights

- The latest research progress in the field of MXene-based neuromorphic computing is reviewed.
- The design strategy of MXene-based neuromorphic devices encompasses multiple factors are summarized, including material selection, circuit integration, and architecture optimization.
- Future development paths for MXene-based neuromorphic computing are discussed, including large-scale manufacturing, stability enhancement, and interdisciplinary integration.

## Graphical abstract

**Figure:**
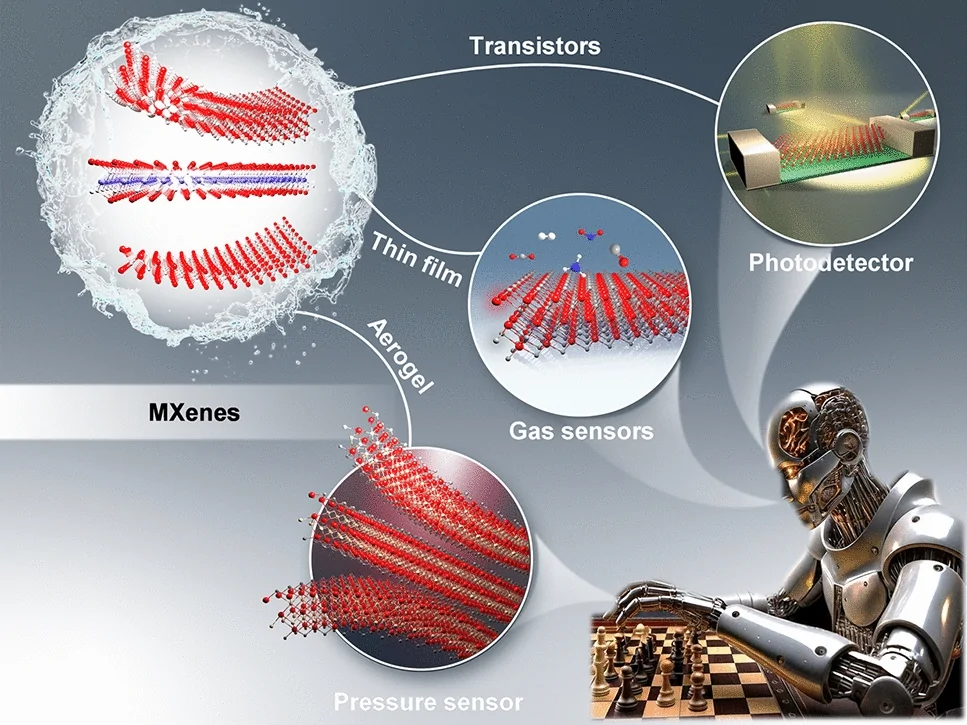

## Introduction

Since their discovery in 2011, two-dimensional transition metal carbides and nitrides (MXenes) have stood out for their unique layered structure and tunable surface chemical properties [1–6], becoming a revolutionary class of materials [3]. Derived from the selective etching of the MAX phase, MXenes features outstanding electrical conductivity [7–10], mechanical flexibility and hydrophilicity, enabling a wide range of applications from energy storage [11, 12] to advanced sensing technologies. Their composition (M_*n*+1_X_*n*_T_*x*_) and surface functional groups (such as –O, –F, –OH) allow for precise modulation of photoelectric, electrochemical and mechanical properties, making them an ideal choice for brain-inspired neuromorphic devices [13–15]. Despite the promising prospects, challenges such as unstable environments, scalability limitations, and interface compatibility in heterogeneous structures have hindered actual deployment. The focus of this article is on MXenes, as their multi-functionality and adaptability address a key gap in next-generation sensors and intelligent systems, providing a way to overcome the limitations of traditional materials through advanced surface engineering and defect modulation.

Neuromorphic computing inspired by biological neural architectures has become increasingly urgent as traditional von Neumann architectures struggle with low energy efficiency and the "memory wall" bottleneck [9, 16–19]. By simulating synaptic plasticity and parallel processing, neuromorphic systems have achieved energy-saving computing and real-time data integration, which is crucial for applications such as robotics and the Internet of Things. However, the existing memory materials usually lack the tunability and stability required for scalable implementation. MXenes, with its metallic conductivity, ion insertion capability and compatibility with flexible substrates, offers a transformative opportunity to bridge this gap. Their ability to simulate synaptic functions (for example, long-duration enhancement) and integrate with multimodal sensors meets the demand for adaptive, low-power neuromorphic hardware. This synergy prompts us to focus on Mxene-based neuromorphic devices, as their unique electronic states and interface dynamics can redefine the paradigms of intelligent sensing and computing.

**Fig. 1:**
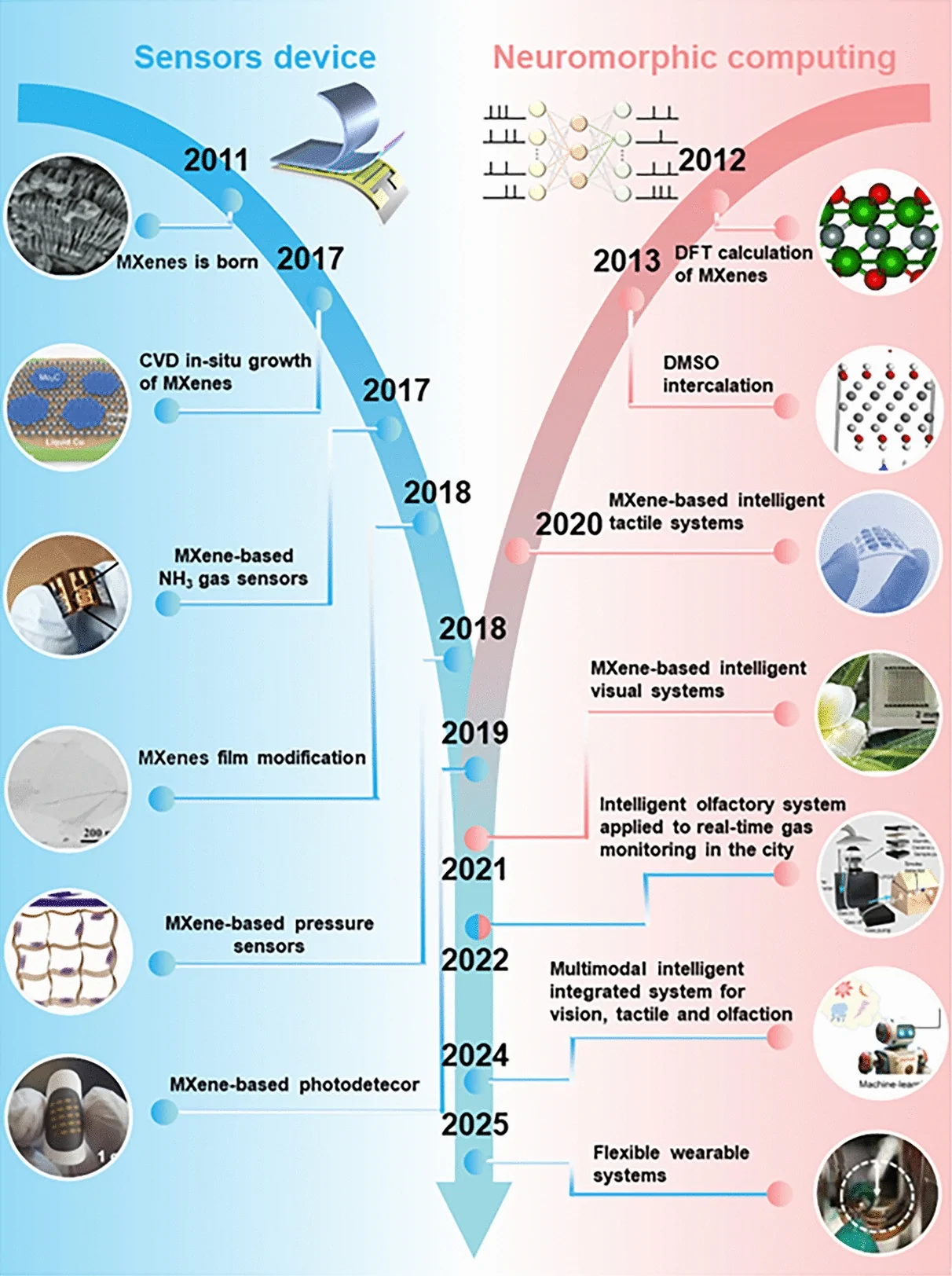
Timeline of the development of MXenes. MXenes were discovered in 2011. Reproduced with permission [3]. Copyright 2011, Wiley‐VCH. The development of the MXenes principle and various MXenes preparation methods was verified by Density Functional Theory (DFT) (Reproduced with permission [29]. Copyright 2012, Wiley‐VCH.), and MXenes-based pressure sensors, photodetectors, and gas sensors were born in turn after 2018. (Reproduced with permission [26]. Copyright 2013, Springer Nature. Reproduced with permission [30]. Copyright 2017, Wiley‐VCH. Reproduced with permission [31]. Copyright 2018, Wiley‐VCH. Reproduced with permission [27]. Copyright 2018, American Chemical Society. Reproduced with permission [23]. Copyright 2019, Wiley‐VCH. Reproduced with permission [20]. Copyright 2017, American Chemical Society). Combined with neural network algorithms, intelligent tactile systems, visual systems, olfactory systems, multi-modal integrated systems and wearable systems are further born (Reproduced with permission [24]. Copyright 2020, Springer Nature. Reproduced with permission [22]. Copyright 2021, Wiley‐VCH. Reproduced with permission [21]. Copyright 2022, Wiley‐VCH. Reproduced with permission [25]. Copyright 2024, Wiley‐VCH. Reproduced with permission [28]. Copyright 2025, Wiley‐VCH)

This paper systematically explores the evolution of MXenes from basic material properties to neuromorphic intelligent systems. Figure 1 illustrates the developmental trajectory of MXenes since their discovery in 2011, encompassing advancements from sensor applications to the integration within intelligent systems. First, we analyzed the structural and functional attributes of MXenes, emphasizing their roles in photodetectors, pressure sensors and gas sensors. MXene-based neuromorphic devices can simulate biological sensory processing capability for constructing emerging visual, tactile and olfactory systems. Finally, we have addressed the challenges in wearable integration, large-scale production and stability, and at the same time proposed future development directions, such as the hybrid CMOS-MXene architecture and the third-generation peak neural network. By placing MXenes within the framework of neuromorphic computing, this work aims to accelerate their transition from laboratory breakthroughs to real-world AI-driven applications.

## Overview of MXenes

### Structure and Classification

**Fig. 2:**
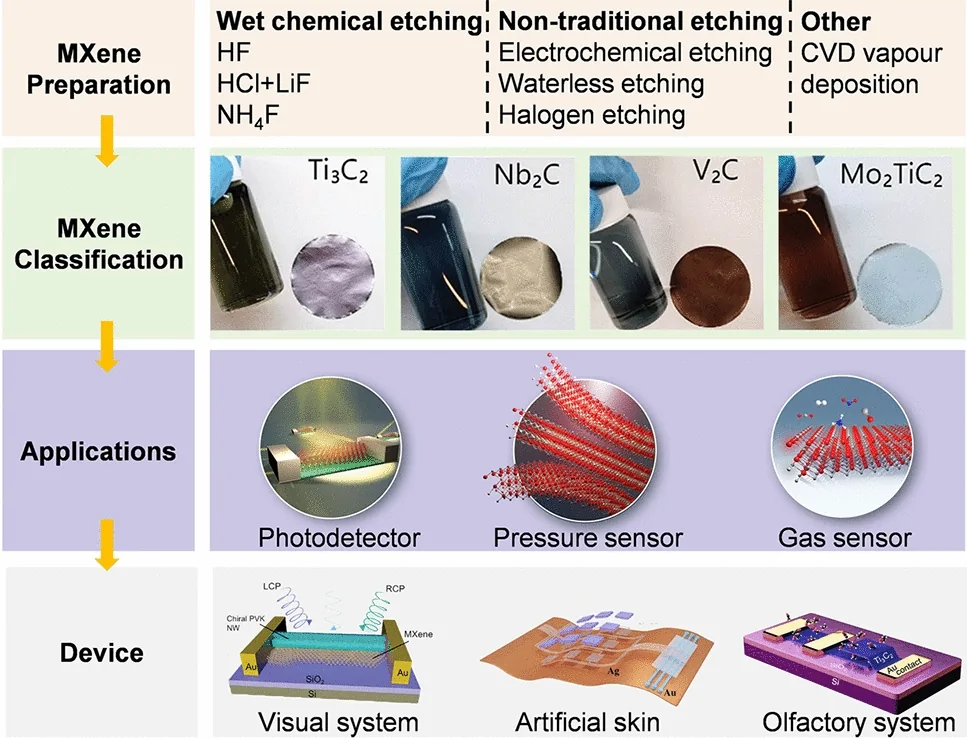
Preparation, types, applications, and intelligent devices of MXenes. Among them, the preparation methods of MXenes include wet chemical etching, non-traditional etching and other. MXenes include TigC_2_, Nb_2_C, V_2_C, and Mo_2_TiC_2_, among others. Reproduced with permission [59]. Copyright 2021, Wiley‐VCH. The smart devices prepared by MXenes include visual system, artificial skin, and olfactory system. Reproduced with permission [56]. Copyright 2024, Wiley‐VCH. Reproduced with permission [57]. Copyright 2024, Elsevier. Reproduced with permission [58]. Copyright 2021, American Chemical Society

The "A" layers of MAX phase precursors, which are usually elements from groups IIIA or IVA, are chemically etched to create MXenes, a family of newly discovered two-dimensional transition metal carbides or nitrides. Their crystal structure is similar to that of the MAX phase, belonging to the hexagonally close-packed (HCP) structure with P63/mmc space group symmetry. In this structure, the “M” sites are composed of closely packed layers of transition metal atoms, while the “X” atoms occupy the octahedral sites between these metal atom layers. The reported MXenes can include “M” elements such as Ti, V, Nb, Mo, Cr, Zr, Hf, Sc, Ta, W, and Y. Notably, Cr, Sc, W, and Y are often reported to act in conjunction with other metals and have only been documented under specific conditions in ortho-xylene or meta-xylene [32]. As research advances, the methods for synthesizing MXenes have become increasingly diverse, as illustrated in Fig. 2, including various processes such as chemical etching with HF, LiF, and NH_4_F, anhydrous etching, and direct growth via chemical vapor deposition (CVD).

**Fig. 3:**
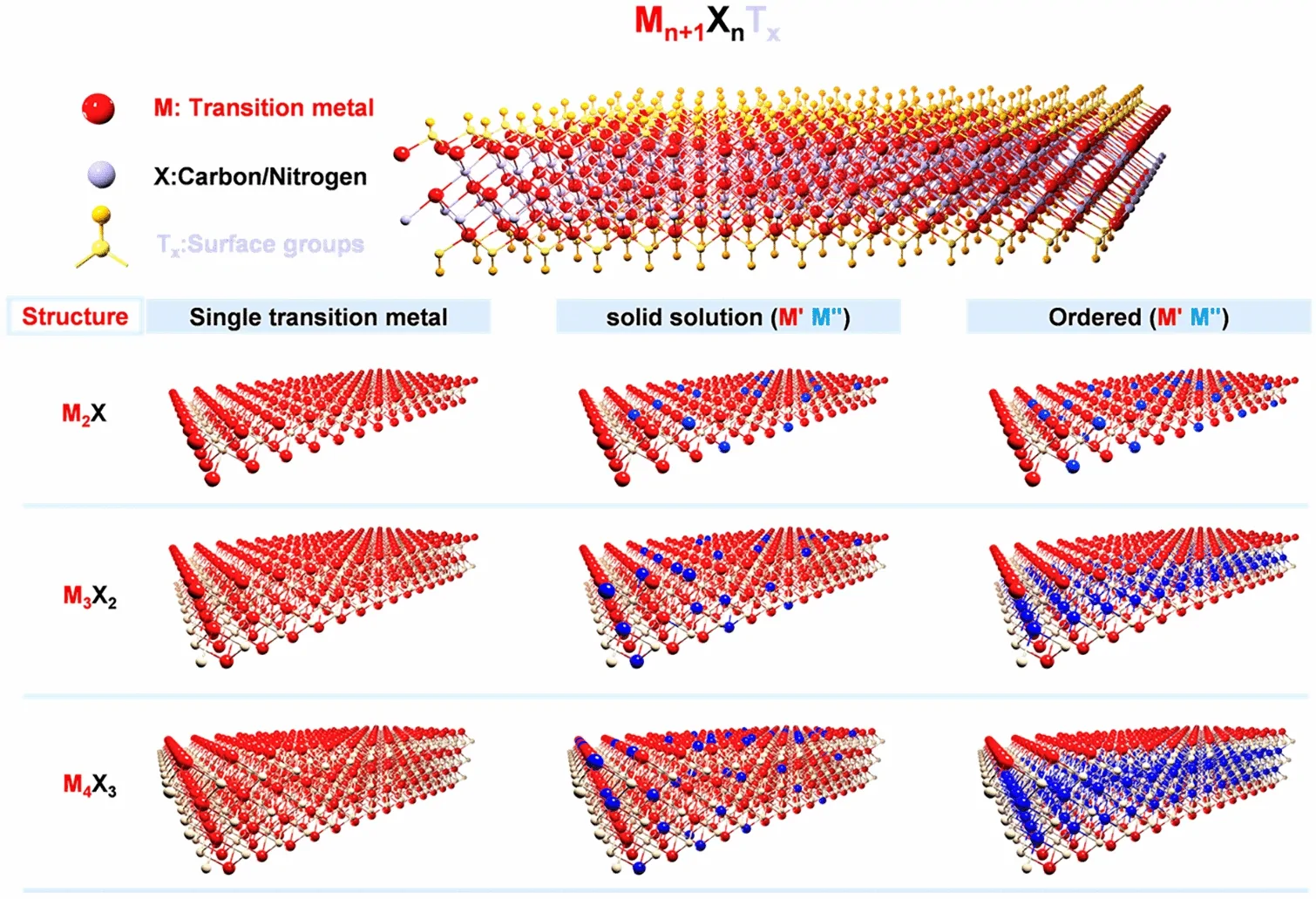
Presents a schematic diagram of the MXene structure. The general formula for binary MXenes is M_*n*+1_X_*n*_T_*x*_, where M denotes early transition metals, *X* represents carbon and/or nitrogen, and Ta refers to the surface terminations of the outer metal layers. The value of *n* can vary between 1 and 4, depending on the number of transition metal layers and carbon and/or nitrogen layers within the MXene structure. For instance, Ti_2_CT_*x*_ (*n* = 1), Ti_3_C_2_T_*x*_ (*n* = 2), Nb_4_C_3_T_*x*_ (*n* = 3), and (Mo,V)_5_C_4_T (*n* = 4) represent different examples. The sites in MXenes can be occupied by one or more transition metaatoms, forming solid solutions or ordered structures. Ordered double transition metal MXenes may exhibit in-plane order, in-plane vacancies, or out-of-plane order with one layer of M" transition metal situated between two layers of M'transition metals, or two layers of " transition metals sandwiched between two layers of 'transition metals. Furthermore, other arrangements, such as a single or triple layer of" situated between ' layers (as illustrated at the bottom of the figure), may also occur in the 5 × 4 structure. The light-colored schematic at the bottom of the figure indicates predicted structures such as high-entropy MXenes and higher-order single or o-MXenes that have yet to be experimentally verified

M_*n*+1_X_*n*_T_*x*_ is the general formula for MXenes, where M stands for transition metals (such as Ti, Zr, Hf, V, and Nb), *X* for carbon and/or nitrogen, and T for terminal groups (such as =O, –OH, and –F). As shown in Fig. 3, over 30 different MXenes, such as Ti₃C₂, Nb₂C, V₂C, and Mo₂TiC₂, have been successfully synthesized [33]. For example, the chemical formula of titanium carbide MXene with two layers of transition metals (*n* = 1) and random terminations is Ti_2_CT_*x*_, where the fully oxidized or fully chlorinated variants are denoted as Ti_2_CO_2_ and Ti_2_CCl_2_, respectively [34]. If two randomly distributed transition metals occupy the M sites in the MXene structure and form a solid solution, the formula can be expressed as (M′, M′)_*n*+1_X_*n*_T_*x*_, where M′ and M′ represent two distinct metals (e.g., (Ti, V)_2_CT_*x*_). For specific metal compositions, the concentration of each element is indicated as a decimal (e.g., (Ti_0.66_V_0.34_)_2_CT_*x*_). When these two metals are arranged in an ordered manner within the plane and form alternating chains of M' and M' atoms within the same M layer, the resulting MXene structure is referred to as i-MXene. To date, all known i-MXenes conform to the formula (M'_4/3_ M'_2/3_)Xₜₓ, where the concentration of each element is expressed in fractional form. In most i-MXenes, M'' atoms can be selectively etched to create ordered vacancies, resulting in i-MXenes with the formula M'_4/3_X_t_ₓ, previously referred to as M'_1.33_X_t_ₓ. Additionally, M' and M' atoms can also be situated on independent atomic planes with out-of-plane order, known as o-MXenes, where M' atoms constitute the inner metal layer while M' atoms reside in the outer layer. Currently, known o-MXenes have two formulas: (M'_2_ M'')X_2_T_*x*_ and (M'_2_ M''_2_)X_3_T_*x*_. Moreover, all MXenes can be produced in the form of multilayer particles (ml-MXene) or as monolayer sheets (d-MXene) [32].

### Fundamental Properties

#### Optical Properties

Due to their broad range of absorption band gaps and suitable energy level distributions, MXenes exhibit substantial appeal in optical applications, such as photothermal and photoelectrochemical devices. Essentially, the optical responses of MXenes (including light absorption and transmittance) are primarily determined by their structural and electronic characteristics. Berdiyorov has validated the crucial function of surface termination in the optical and dielectric characteristics of TiC_2_T_*x*_ using density functional theory (DFT) computations [35]. For instance, Ti_3_C_2_O_2_, treated with –F and –OH, demonstrates lower light absorption and reflection characteristics compared to the pristine Ti_3_C_2_O_2_. However, in the ultraviolet region, surface termination with –F, =O, and –OH can enhance the reflectivity of Ti_3_C_2_, thereby endowing it with excellent UV resistance. Gao et al. used Z-scan and transient absorption methods to study the broadband nonlinear optical responses and carrier relaxation properties of Mxenes [36]. They found that the multiphoton absorption effects of these materials might result in saturable absorption and reverse saturable absorption phenomena. Furthermore, Bai et al. revealed that the optical band gap of Ti_2_CO_2_ is approximately 1 eV, while in the out-of-plane direction, this band gap can be expanded to 2.3 eV, a phenomenon also observed in the metallic Ti_2_CF_2_ and Ti_2_C_2_O_2_ [37]. These studies indicate that the optical properties of MXenes can be effectively modulated through surface functionalization, showcasing their immense potential in the field of light energy capture and utilization.

#### Mechanical Properties

The mechanical properties of MXenes are critical parameters for their practical applications, such as in pressure sensors and artificial skin, and are influenced by various factors. Firstly, the surface terminal groups significantly affect the mechanical strength of MXenes. Dai et al. proposed an interface engineering approach that combines aramid (Kevlar) nanofibers with borate ions to enhance the mechanical characteristics of Mxenes [38]. The optimized MKB fibers exhibited a mechanical strength that was 3.86 times greater than that of the original MXene nanosheets, demonstrating a high stress characteristic of 242.6 MPa. The improved mechanical flexibility can be attributed to the surface termination, which alleviates the collapse of the titanium layers. Furthermore, studies indicate that MXenes terminated with functional groups (e.g., =O) exhibit higher elasticity compared to bare Mxenes [39]. Fu et al. analyzed the impact of functional groups on mechanical strength using DFT [40], revealing that terminated Ti_3_C_2_T_*x*_ outperforms the original Ti_3_C_2_ in terms of in-plane Young's modulus (228 N m⁻^1^), with functionalized Ti_3_C_2_ achieving the highest Young's modulus value of 347 N m⁻^1^, surpassing other two-dimensional materials such as WS_2_ (approximately 177 GPa) and GO (approximately 340 N m⁻^1^) [41]. This phenomenon is attributed to the strong bond interactions between Ti and O within MXenes, which weaken the Ti–Ti bond interactions, thereby enhancing mechanical performance. Additionally, the thickness of MXenes may also exert a potential influence on mechanical stiffness, particularly in the context of M–M bonding interactions.

#### Electronic Properties

MXenes' composition, structure, and the chemical makeup of their surfaces and interlayers all have a major impact on their physical and (electro) chemical properties. Most MXenes have electrical transport behavior similar to that of metals, with a linear decrease in resistance with increasing temperature [42, 43]. However, some MXenes display semiconducting qualities as a result of the negative connection between resistivity and temperature caused by changes in the kind of transition metal and the structure of the “M” site in Mxenes [44, 45], such as Mo_2_CT_*x*_, Nb_2_CT_*x*_, and V_2_CT_*x*_ [46, 47]. In contrast, Ti_3_C_2_T_*x*_ MXenes demonstrate metallic conductivity, while the substitution of Ti layers by Mo to form Mo_2_TiC_2_T_*x*_ results in semiconducting behavior [48].

Anasori et al. altered the density of states (DOS) of MXenes by modifying the structure of the “M” sites and enhanced the antiferromagnetic properties of MXenes through changes in the outer transition metals (M′′) [45]. In certain simulated MXenes, the central M′′ layer is in a non-spin-polarized state, while the surface M’ layer experiences band splitting of its 3*d* orbitals under the influence of an octahedral crystal field, thus defining the magnetic ordering of MXenes [49].

The surface terminations allow for customizable electronic characteristics by modulating the DOS and changing the fermi level, in contrast to conventional metals [48]. Significant changes in work function have been expected along with the bandgap expansion and the switch from metallic to semiconducting behavior for a number of MXenes, with the exception of Ti_3_C_2_T_*x*_ [43, 50]. While superconductivity has been seen in Nb_2_CT_*x*_ (where T_*x*_ = Se, S, or NH), no superconducting transition was detected in non-terminated or O-terminated multilayer Nb_2_C [51]. It has been noted that as the number of layers increases, the bandgap gradually decreases, primarily due to weak interlayer coupling effects [16]. Zhou et al. [52] conducted a systematic study of semiconductor M_2_CO_2_ MXenes (M=Ti, Zr, or Hf) with varying layer numbers, revealing that the bandgaps of bilayer Ti_2_CO_2_, Zr_2_CO_2_, and Hf_2_CO_2_ (0.039, 0.729, and 0.824 eV, respectively) are all smaller than those of single-layer M_2_CO_2_ (0.281, 0.950, and 1.020 eV), which is mainly attributed to the rise in electronic states toward the Fermi level due to interlayer interactions in multilayer M_2_CO_2_.

Moreover, thicker MXenes (e.g., M_3_X_2_T_*x*_ and M_4_X_3_T_*x*_) are generally considered metallic materials under any termination state [29]. Intercalants commonly used between the layers can also impact the electronic properties of multilayer films and thin films [44]. The incorporation of large organic cations, such as dimethyl sulfoxide (DMSO), between MXene sheets increases the interlayer spacing, hindering electron hopping and consequently reducing the conductivity of the films [26, 53]. In contrast, intercalated alkali metal cations often maintain smaller interlayer spacing and higher conductivity in Mxenes [54, 55].

### Applications and Intelligent Devices

As of now, MXenes have demonstrated significant potential in the field of sensors, primarily attributable to their exceptional electronic, optical, and mechanical properties, as well as their diverse terminal groups and tunable active sites. Ma et al. developed an aerogel piezoresistive sensor in conjunction with graphene, which can effectively capture signals as low as 10 Pa, thereby enabling the clear identification of adult pulses. This sensor outperforms conventional sensors based solely on reduced graphene oxide (rGO) or Mxene [27]. Velusamy et al. fabricated Mo_2_CT_*x*_ films that fully leverage the unique optical properties of MXenes, achieving breakthroughs in applications such as photodetectors for the first time using photoelectronic spectroscopy techniques [23]. The diverse functional groups and electronic characteristics of MXenes provide significant insights for the field of gas sensing. Lee et al. developed a Ti_3_C_2_T_*x*_ MXene gas sensor capable of detecting various gases, including ethanol, ammonia, and acetone, at room temperature, marking an important advancement in the operation of gas sensors at ambient conditions [20]. Furthermore, with the rise of neuromorphic computing, the integration of MXenes with various sensors has led to the emergence of a range of MXene-based neuromorphic vision systems [22, 56], artificial skin [24, 57], olfactory systems [21, 58], and multimodal intelligent integrated systems [25].

## MXenes-Based Sensors

### Photodetectors

MXene, as a two-dimensional transition metal carbon/nitride, exhibits unique photoelectric properties: Its band gap can be flexibly regulated by surface terminal groups (such as –O, –F, –OH) (0.1–2.3 eV), supports wide-spectrum light absorption from ultraviolet to near-infrared (325–808 nm), and has high carrier mobility (~ 10^3^ cm^2^ V⁻^1^ s⁻^1^) and local surface plasmon resonance effect (LSPR). Significantly enhance the light-matter interaction. In addition, the adjustable range of the work function of MXene is wide (1.81–6.15 eV), and the charge extraction efficiency can be optimized through interface energy level matching (such as when combined with semiconductor heterojunctions). MXenes exhibit exceptional potential as active light-absorbing layers in photodetectors due to their tunable bandgap, broadband light absorption (from UV to near-infrared), and high carrier mobility. These properties stem from their unique electronic structure and surface terminations, which enable efficient photon-to-electron conversion [60]. For instance, Ti_3_C_2_T_*x*_ MXenes demonstrate strong light-matter interactions owing to their localized surface plasmon resonance and adjustable work function (1.81–6.15 eV), allowing precise alignment with adjacent semiconductor layers to minimize energy barriers for charge extraction [61]. Its high conductivity and tunable work function ensure low energy barriers for hole injection, facilitating the application of MXenes in devices, such as photodetectors [62], OLEDs [63], and photonic diodes [64].

**Fig. 4:**
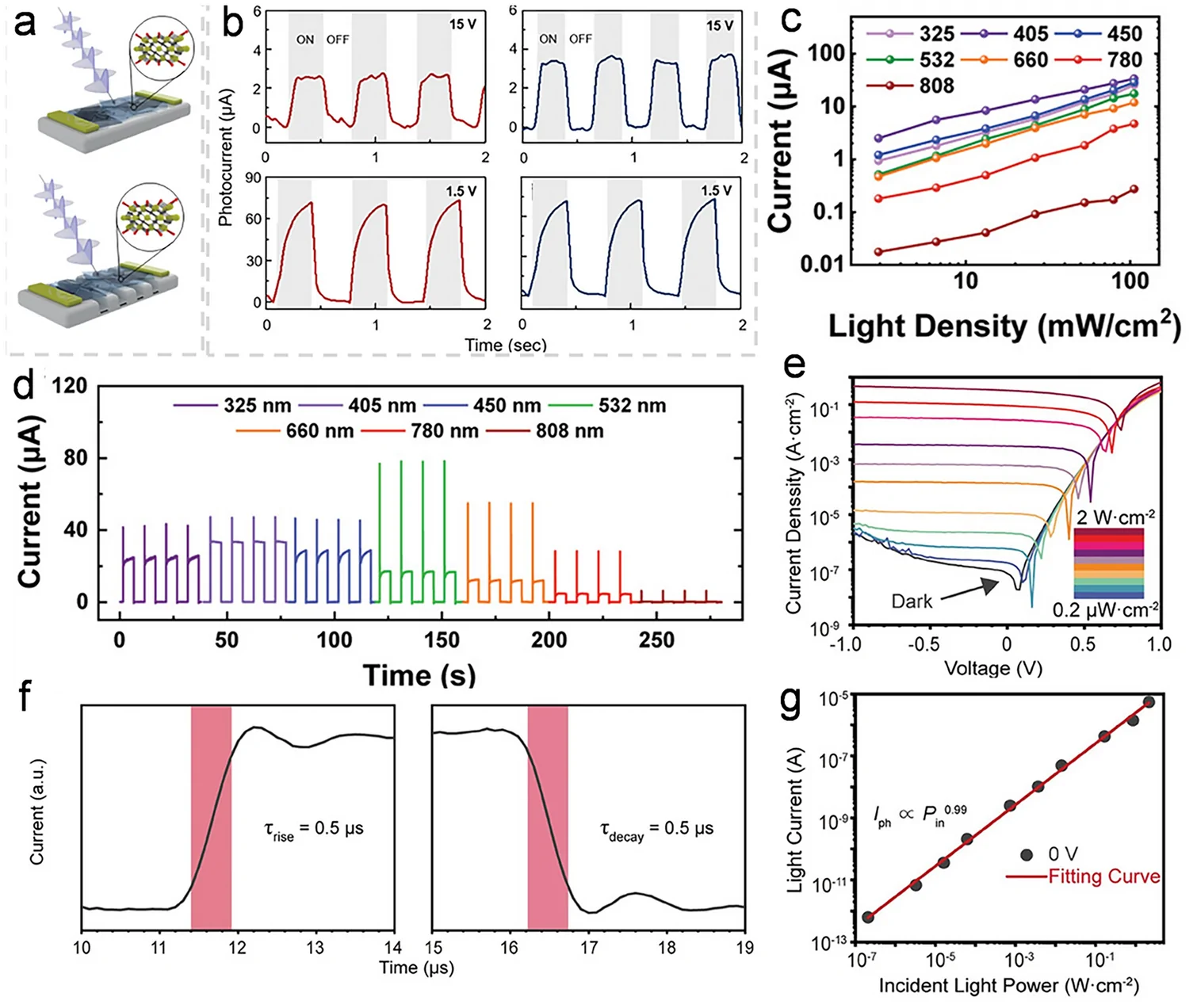
Applications of MXenes in optoelectronics and sensors: **a** A schematic diagram of the MXenes photodetector structure. **b** Test results for the switching characteristics of the pulsed current. Reproduced with permission [62]. Copyright 2024, Wiley‐VCH. **c**, **d** Performance evaluation of MXenes under different laser wavelengths (325, 405, 450, 532, 660, 780, and 808 nm). Reproduced with permission [65]. Copyright 2024, Wiley‐VCH. **e** Dark current test data. **f** Response and recovery time testing of the Ti_3_C_2_T_*x*_/PbS quantum dot photodiode. **g** Analysis of the linear relationship between photocurrent and optical power. Reproduced with permission [66]. Copyright 2024, Wiley‐VCH

Figure 4a illustrates a photodetector structure where MXene serves as the active layer, leveraging its high photoresponsivity and fast carrier dynamics [62]. Under repeated illumination conditions with bias voltages of 15 and 1.5 V, it exhibits clear optical pulse current switching characteristics (Fig. 4b), indicating that MXenes has excellent performance and good reliability and reversibility in photodetectors [62]. Utilizing laser light sources with wavelengths of 325, 405, 450, 532, 660, 780, and 808 nm, MXenes exhibit excellent optoelectronic output (Fig. 4c). In continuous multiple pulse testing, MXenes generate maximum photocurrent when stimulated by a 405 nm laser (Fig. 4d) [65]. Figure 4e demonstrates that the dark current density of the device, tested at a bias voltage of − 0.5 V under dark conditions, is 0.2 µA cm⁻^2^, indicating that MXenes devices possess an extremely low dark current. This lower dark current contributes to enhanced sensitivity in photodetectors, allowing for more effective detection of weak optical signals [66]. For photodetectors, a short response time is crucial, as it enables the device to rapidly sense changes in light intensity and respond accordingly. The response time is determined by the recombination of excess charge carriers and the time required to eliminate interlayer defects [67]. For example, Di et al. achieved sub-microsecond response times in Ti₃C₂T*ₓ*/PbS quantum dot heterostructures by eliminating deep-level traps through solution-processed interfacial engineering, effectively suppressing dark current while maintaining high conductivity (Fig. 4f) [66]. Figure 4g illustrates the linear relationship between photocurrent and optical power in a self-powered state (I__ph_ ∝ P__in_⁰⁹⁹) [66]. The linear dynamic range (LDR) is another critical metric for photodetectors, characterizing the consistency of the detector's response to variations in light intensity.

Table 1 summarizes the performance metrics of three representative MXene-based photodetectors under different material configurations. The Ti_3_C_2_T_*x*_ MXene photodetector operates at a wavelength of 1064 nm, achieving a response time of 0.207 ms and a photoresponsivity of 0.41 A W^−1^. The ITO/Ti_3_C_2_T_*x*_/MAPbI_3_/Au heterostructure demonstrates broadband detection capabilities spanning 325–780 nm, with a response time of 2.8 ms and a photoresponsivity of 0.0014 A W^−1^. Notably, the ZnO/PbS/MXene hybrid device exhibits ultrafast response characteristics (0.5 μs) across a broad spectral range (400–1100 nm), though its photoresponsivity remains unreported in the referenced study. These results highlight the tunability of MXene-based photodetectors, where structural modifications and heterojunction engineering significantly influence wavelength sensitivity, temporal resolution, and photon-to-current conversion efficiency. The Ti_3_C_2_T_*x*_ MXene showcases superior photoresponsivity, while the ZnO/PbS/MXene configuration excels in ultrafast response, underscoring the trade-offs between sensitivity and speed in optoelectronic design.

**Table 1:** Performance comparison of mainstream MXene photodetectors

Materials	Wavelength (nm)	Response time (s)	Photoresponsivity (A W^−1^)	References
Ti_3_C_2_T_*X*_	1064	0.000207	0.41	[62]
ITO/Ti_3_C_2_T_*x*_/MAPbI_3_/Au	325–780	0.0028	0.0014	[65]
ZnO/PbS/MXene	400−1100	0.0000005	–	[66]

### Pressure Sensors

Flexible and wearable self-powered sensors, especially pressure sensors in the fields of clinical health and electronic skin, have been a major area of research interest in recent years [68]. MXenes have gained increasing recognition as critical materials for fabricating high-performance pressure sensors, owing to their exceptional mechanical strength, outstanding electrical and thermal conductivity, and remarkably large specific surface area [69]. However, the range of applications for traditional current-based pressure sensors is limited since they are frequently employed in conjunction with polymer substrates or encapsulating layers, which can cause discomfort during wear (e.g., low air/vapor permeability and mechanical mismatch) [70]. Zheng et al. successfully created a flexible and breathable pressure sensor by using nonwoven textiles as electrodes and coating them with MXene/silver nanowires in order to solve this problem [71]. By ingeniously utilizing the multilayered porous characteristics of MXenes, this sensor not only exhibits high sensitivity but also demonstrates outstanding cycling stability and puncture resistance, capable of detecting various human activities, including subtle pulses, pulse vibrations, and large-scale movements, such as walking and running.

**Fig. 5:**
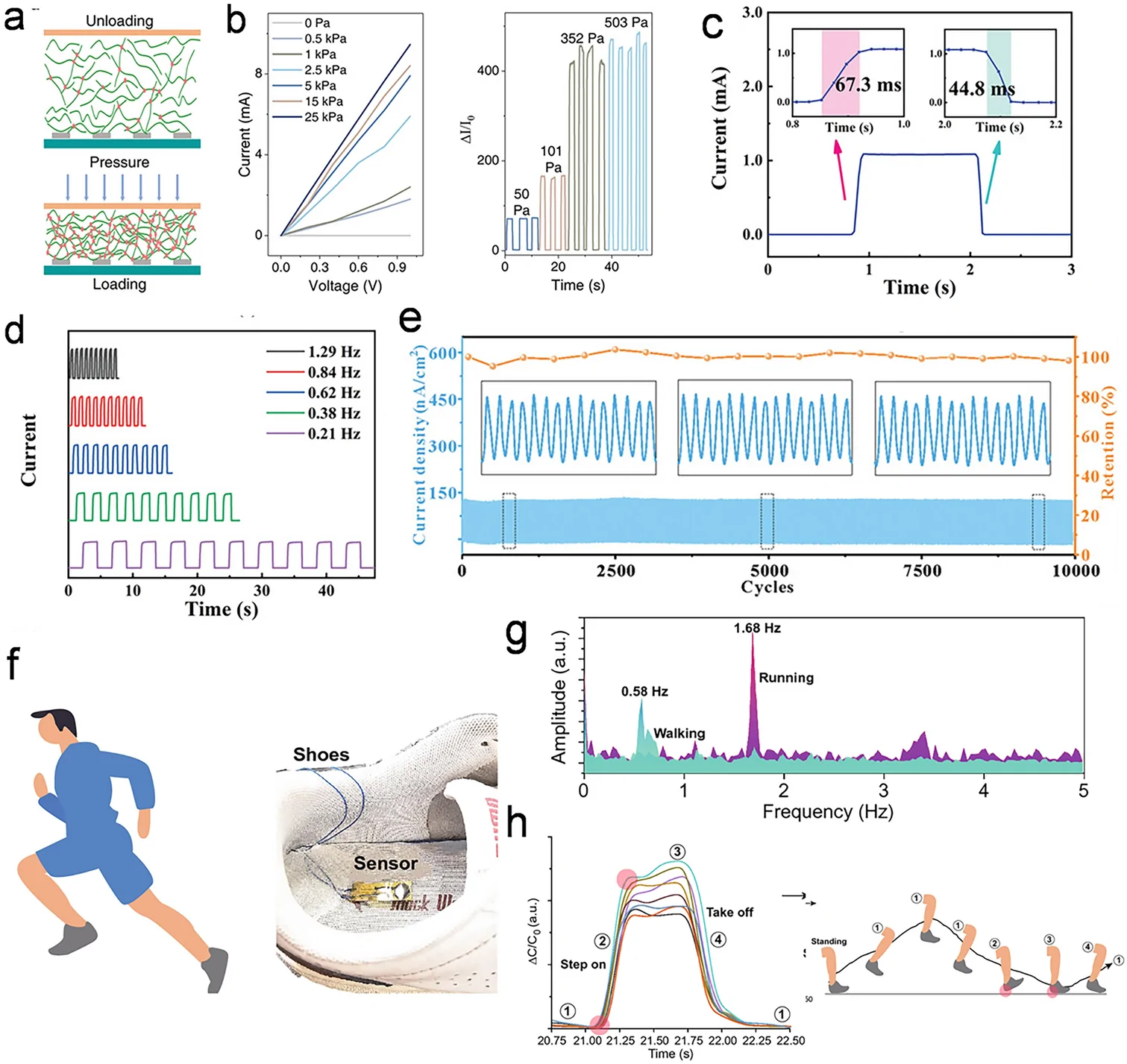
Applications of MXenes in pressure sensors: **a** Schematic diagram illustrating the operating principle of resistive pressure sensors. **b** Current–voltage (I–V) curves within a static pressure range of 0–25 kPa. Reproduced with permission [71]. Copyright 2023, Wiley‐VCH. **c** Response and recovery time test data. **d** Pressure response tests conducted at various frequencies. Reproduced with permission [72]. Copyright 2022, Wiley‐VCH. **e** Stability testing after 10,000 cycles of applied pressure. Reproduced with permission [73]. Copyright 2022, Wiley‐VCH. **f** Schematic representation of MXenes-based pressure sensors in practical applications. **g**, **h** Data collection during human walking and running processes. Reproduced with permission [74]. Copyright 2021, Springer Nature

The operational principle of MXene-based pressure sensors primarily relies on their mechanical properties. When pressure is applied to the device's surface, it induces a change in resistance (as illustrated in Fig. 5a) [71]. Specifically, the sensor undergoes deformation under pressure, resulting in a reduction of the gap between electrodes, which subsequently enhances the contact between MXene materials and significantly decreases the resistance. Within different pressure ranges (0–25 kPa), as shown in Fig. 5b, the current–voltage (I–V) curves of the pressure sensors indicate that the current response sharply increases with the application of pressure, and the relative current change exhibits a monotonically increasing relationship with the external pressure [71]. Notably, within the range of 50–503 Pa, the sensor maintains a stable and nearly linear current response. Due to the exceptional elasticity of materials such as MXene-based aerogels, these pressure sensors exhibit very short response and recovery times (67.3 and 44.8 ms, respectively) (Fig. 5c) [72]. As depicted in Fig. 5d, MXene pressure sensors can accurately identify external pressures of varying frequencies, an important metric for evaluating sensor performance that aids in more precise predictions of human motion speeds (e.g., distinguishing between walking and running) [72].

The MXene self-powered sensor converts mechanical energy into electrical energy through piezoelectric or triboelectric effects, thereby achieving sensing functions without the need for an external power supply. For instance, the MXene aerogel piezoresistive sensor developed by Yue et al. (Fig. 5e) [73], combined with the nano-fluid channel design, is capable of self-generating electricity at a low pressure of 10 Pa. And stably identify the pulse signal. After 10,000 cycles of pressure tests, the current signal of the pressure sensor based on MXene maintained an amplitude similar to its initial state, verifying the stability of the MXene pressure sensor in long-term use and providing a guarantee for commercial applications. This self-powered characteristic stems from the synergistic effect of the high conductivity of MXene and flexible substrates (such as polydimethylsiloxane), generating a potential difference through structural deformation caused by external pressure. Furthermore, the surface terminal groups of MXene (such as -O and -F) can optimize the interfacial charge distribution and further enhance the energy conversion efficiency. Gao et al. installed MXene-based pressure sensors in footwear (Fig. 5f) [74] and analyzed trends in physical activity through fast Fourier transform (Fig. 5g) and the collection of step data (Fig. 5h) [74]. In gait testing, the pressure exerted by the foot on the ground gradually increases from the first to the second step, peaking at the maximum contact point (third step), and finally returning to the original state when the foot is lifted (fourth step). Monitoring the frequency of pressure changes confirms that MXene-based pressure sensors can successfully track human activities such as standing, walking, and running [75]. The self-powered design not only reduces the power consumption of the sensor but also enhances its applicability in wearable devices, such as long-term physiological monitoring or environmental energy harvesting scenarios.

Table 2 systematically compares the performance metrics of four MXene-based pressure sensors with distinct material configurations. The MXene/Ag NW nonwoven fabric sensor exhibits a response/recovery time of 70/81 ms, a detection limit of 1 Pa, and a sensitivity of 770.86 kPa⁻^1^ in the low-pressure range (0–0.75 kPa), which decreases to 5.87 kPa⁻^1^ at higher pressures (10–100 kPa). The MXene/PU composite demonstrates faster response/recovery dynamics (67.3/44.8 ms), a detection limit of 7.8 Pa, and a sensitivity of 509.78 kPa⁻^1^ in the intermediate pressure range (1.7–5.7 kPa). The MXene/CNF configuration, while achieving rapid response (52 ms) and recovery (89 ms), lacks reported sensitivity values but highlights dual-mode current and voltage outputs (− 23.64 nA kPa⁻^1^ and 13.24 μV kPa⁻^1^, respectively). Notably, the MXene/PVA-KOH sensor achieves an ultrahigh sensitivity of 46,730 kPa⁻^1^ at a detection limit of 20 Pa, attributed to its ionic conductive mechanism. These results underscore the critical influence of material composition and structural design on sensor performance, with MXene composites demonstrating trade-offs between sensitivity, dynamic range, and gantemporal resolution. The variability in sensitivity across pressure regimes emphasizes the need for tailored material engineering to optimize sensor functionality for specific applications.

**Table 2:** Performance comparison of mainstream MXene pressure sensors

Materials	Response/recovery time (ms)	Detection limit (Pa)	Sensitivity (kPa^−1^)	References
MXene/Ag NW nonwoven fabric	70/81	1	770.86(0–0.75 kPa), 136.91(1–2 kPa), 43.81(2.5–7.5 kPa), 5.87(10–100 kPa)	[71]
MXene/PU	67.3/44.8	7.8	281.54/(0.2–1.7), 509.78/(1.7–5.7), 66.68/(5.7–20.3)	[72]
MXene/CNF	52/89	–	− 23.64 nA kPa^−1^, 13.24 uV kPa^−1^	[73]
MXene/PVA-KOH	98/70	20	46,730 kPa^−1^	[74]

### Gas Sensors

Gas sensors based on MXenes are capable of effectively detecting volatile organic compounds (VOCs) and non-polar gases, such as ammonia, ethanol, and acetone, at room temperature [76, 77]. This efficiency is primarily attributed to the strong adsorption capabilities of their metallic core channels and surface functional groups toward these gas molecules. Compared to other two-dimensional material-based gas sensors, Ti_3_C_2_T_*x*_-based gas sensors demonstrate a significantly improved signal-to-noise ratio at room temperature and exhibit higher sensitivity in detecting volatile organic compounds [20, 78]. MXenes have thus emerged as ideal materials for high-performance wearable gas sensors [77].

**Fig. 6:**
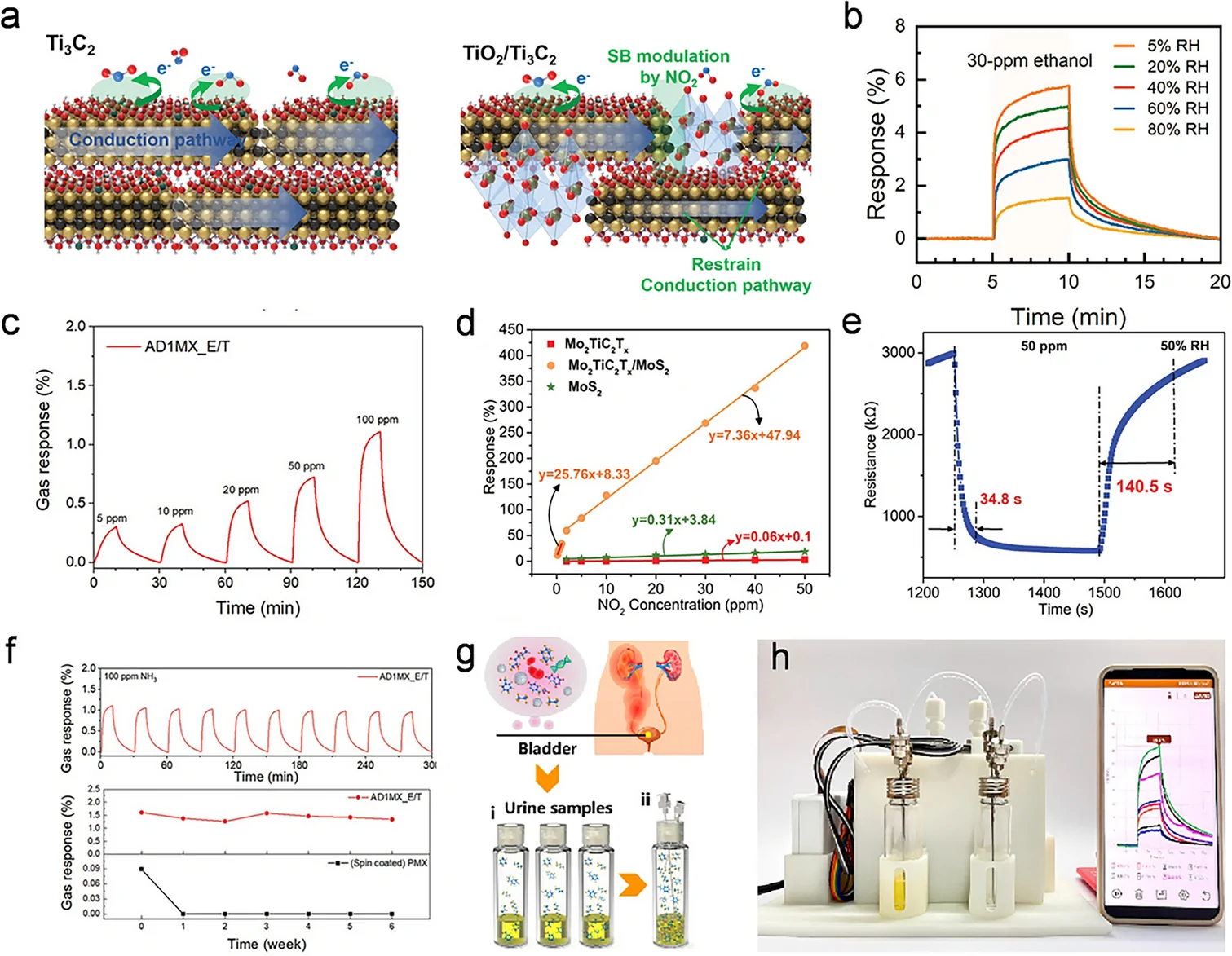
Applications of MXenes in gas sensors: **a** Role of MXenes heterojunction engineering in gas sensing mechanisms. Reproduced with permission [79]. Copyright 2020, Wiley‐VCH. **b** Dynamic response curves to 30 ppm ethanol under varying humidity conditions. Reproduced with permission [85]. Copyright 2022, American Chemical Society. **c** Dynamic response curves under changes in gas concentration. Reproduced with permission [86]. Copyright 2024, Wiley‐VCH. **d** Linear fitting curves for different gas concentrations of Mo_2_TiC_2_T_*x*_, MoS_2_, and Mo_2_TiC_2_T_*x*_/MoS_2_ **e** Response and recovery time testing. Reproduced with permission [87]. Copyright 2022, Wiley‐VCH. **f** Results of long-term stability testing. Reproduced with permission [86]. Copyright 2024, Wiley‐VCH. **g** Schematic diagram of urine sample collection. **h** Design and application of a portable volatile gas detection platform for urine. Reproduced with permission [88]. Copyright 2022, American Chemical Society

Figure 6a illustrates the working mechanism of MXenes in gas sensing [79]. The primary sensing mechanisms include surface charge transfer and Schottky barrier modulation. When gas molecules adsorb onto the surface of the material, the resistance of MXenes changes [80–83]. During the surface charge transfer process, the interaction between gas molecules and MXenes can result in either an increase or decrease in resistance, depending on the type of charge carriers in the semiconductor and the electronic properties of the gas molecules. For instance, strongly reducing gases can lower the barrier, significantly facilitating electron transport, thereby producing a large gas sensing response when holes are the predominant charge carriers [83].

In the design of gas sensors, humidity is one of the main interfering factors affecting gas sensitivity. Under high humidity conditions, water molecules can cover the surface of the material and occupy the active sensing channels, leading to a reduction in sensor response [84]. To address this issue, Chen et al. successfully developed humidity-interference-resistant gas sensors by surface treating MXenes with fluoroalkyl silane (FOTS) [85]. As shown in Fig. 6b, the sensor exhibits minimal response variation to 30 ppm ethanol across a relative humidity range of 5%–80% [85].

By testing the dynamic response curves of different concentrations of gas (Fig. 6c) [86] and performing linear fitting (Fig. 6d) [87], the limit of detection (LOD) for the gases can be predicted. Additionally, the short response and recovery times indicate the high sensitivity of the sensor (Fig. 6e) [87]. Although many MXenes modification methods can enhance gas sensing performance, they often compromise stability. Kim et al. achieved significant stability improvements—up to six weeks—by functionalizing MXenes with ADOPA and rapidly fabricating nanoscale films (Fig. 6f) [86]. Thanks to the excellent performance of MXenes-based gas sensors, these sensors can provide early non-invasive clinical warnings of diseases by detecting volatile gases in human breath or urine. Ding et al. developed a portable urine detection platform combining gas flow control with a signal reading module. As illustrated in Fig. 6g, h, this platform integrates with smartphones to facilitate the visualization of urine information, thereby enabling disease warning functionalities [88].

Table 3 systematically compares the performance indicators of MXene gas sensors configured with four different materials. The response/recovery time of the TiO_2_/Ti_3_C_2_ sensor is 70/81 s and the response rate is 20%. The Ti_3_C_2_T_*x*_-F structure has better kinetic properties, with a response/recovery time of 39/139 s, a variable response range (2.1%–14.1%), and a detection range of 5–12 ppm. The AD-MXene sensor achieves a response of 1.0% and a wide detection range of 1–100 ppm. It is worth noting that the Mo_2_TiC_2_T_*x*_/MoS_2_ heterostructure has excellent performance, with a response/recovery time of 34.8/140.5 s, a response rate as high as 415.8%, and a detection range of 0.2–50 ppm. These results emphasize the crucial influence of material composition and heterojunction engineering on sensor performance. Mo_2_TiC_2_T_*x*_/MoS_2_ exhibits excellent sensitivity due to the synergistic interfacial charge transfer effect. The variations in detection range and response amplitude emphasize the importance of tailoring material designs for specific gas-sensitive applications, such as trace-level detection or broad-spectrum environmental monitoring.

**Table 3:** Performance comparison of mainstream MXene gas sensors

Materials	Response/recovery time (s)	Response (%)	Detection range (ppm)	References
TiO_2_/Ti_3_C_2_	70/81	20	–	[79]
Ti_3_C_2_T_*x*_-F	39/139	2.1–14.1	5–12	[85]
AD-MXene	–	1.0	1–100	[86]
Mo_2_TiC_2_T_*x*_/MoS_2_	34.8/140.5	415.8	0.2–50	[87]

## Transition of Sensors to Neuromorphic Devices

In recent decades, significant advancements have been made in traditional von Neumann computer architectures; however, as Moore's Law approaches its limits, this architecture faces challenges in overcoming the von Neumann bottleneck [89, 90]. In this context, the demand for capabilities that meet the requirements of future high-performance artificial intelligence systems has become increasingly daunting. Consequently, the need to develop novel computational paradigms that offer higher energy efficiency and processing power is more urgent than ever. Neuromorphic computing, as an emerging computational paradigm, provides a potential solution to the challenges posed by traditional computer architectures in the era of flexible electronics by mimicking the operational mechanisms of biological nervous systems [91–93]. Inspired by the complexity of the human brain, neuromorphic computing aims to effectively integrate storage and computation, adhering to the energy-saving principles of biological neural systems. Meanwhile, the combination of self-powered MXene sensors and neuromorphic computing provides a new direction for edge intelligence. For example, many researchers have integrated the MXene piezoelectric sensor with the spiking neural network (SNN), achieving self-powered real-time processing of gait signals. The MXene sensor in the system directly converts mechanical stimuli into electrical pulse signals and performs spatiotemporal feature extraction through SNN, reducing the reliance on the central processing unit. This self-powered—neuromorphic collaborative architecture significantly reduces the overall energy consumption of the system, providing a paradigm for distributed perceptual computing in Internet of Things devices. This paradigm holds the promise of bringing about profound transformations in the fields of artificial intelligence and cognitive computing [94–96]. In recent years, neuromorphic computing has been implemented across various application domains, ranging from robotic vision to real-time recognition in multimodal integration, demonstrating its significant practical potential and broad prospects [97, 98].

Recent advances in MXene-based memristors have demonstrated their potential to bridge the gap between data storage and neuromorphic computing. MXenes' tunable electronic properties, such as controllable surface terminations and defect engineering, enable precise modulation of resistive switching (RS) behavior, which is critical for non-volatile memory and synaptic weight simulation. For instance, Ti_3_C_2_T_x_ MXene memristors exhibit stable bipolar RS characteristics with low operating voltages (< 1 V) and high ON/OFF ratios (> 10^3^), mimicking biological synaptic plasticity for both short-term and long-term potentiation (LTP) [99, 102]. The intercalation of ions (e.g., Li⁺, Na⁺) between MXene layers facilitates dynamic conductance modulation, enabling analog switching suitable for in-memory computing architectures. Furthermore, MXene/polymer heterostructures have achieved multi-level storage capabilities by leveraging interfacial charge trapping effects, which are essential for high-density neuromorphic systems [100, 138]. Integrating MXene memristors with sensor arrays could enable real-time signal processing and storage at the edge, significantly reducing energy consumption and latency in intelligent systems. Future research should focus on optimizing MXene memristor endurance (> 10^6^ cycles) and scalability for large-scale integration with spiking neural networks (SNNs).

### Neuromorphic Visual Systems

Inspired by simulation studies and the ongoing advancements of MXenes in the field of high-performance optoelectronic detectors, numerous researchers are actively exploring new methods to expand the applications of MXenes in optoelectronics. With the development of neuromorphic computing, MXenes have been successfully utilized as photosensitive materials in transistors, facilitating their widespread application in visual neuromorphic contexts. The interlayer ion intercalation ability of MXene (such as Li⁺, Na⁺) and its tunable electron state density (DOS) enable it to simulate the ion migration and charge storage behavior of biological synapses. Furthermore, its mechanical flexibility provides unique advantages for the design of wearable neuromorphic devices. By constructing an array of MXenes-based optoelectronic detectors and integrating them with neuromorphic computing, intelligent applications such as image learning, optoelectronic signal conversion, and trained recognition have been achieved [99].

**Fig. 7:**
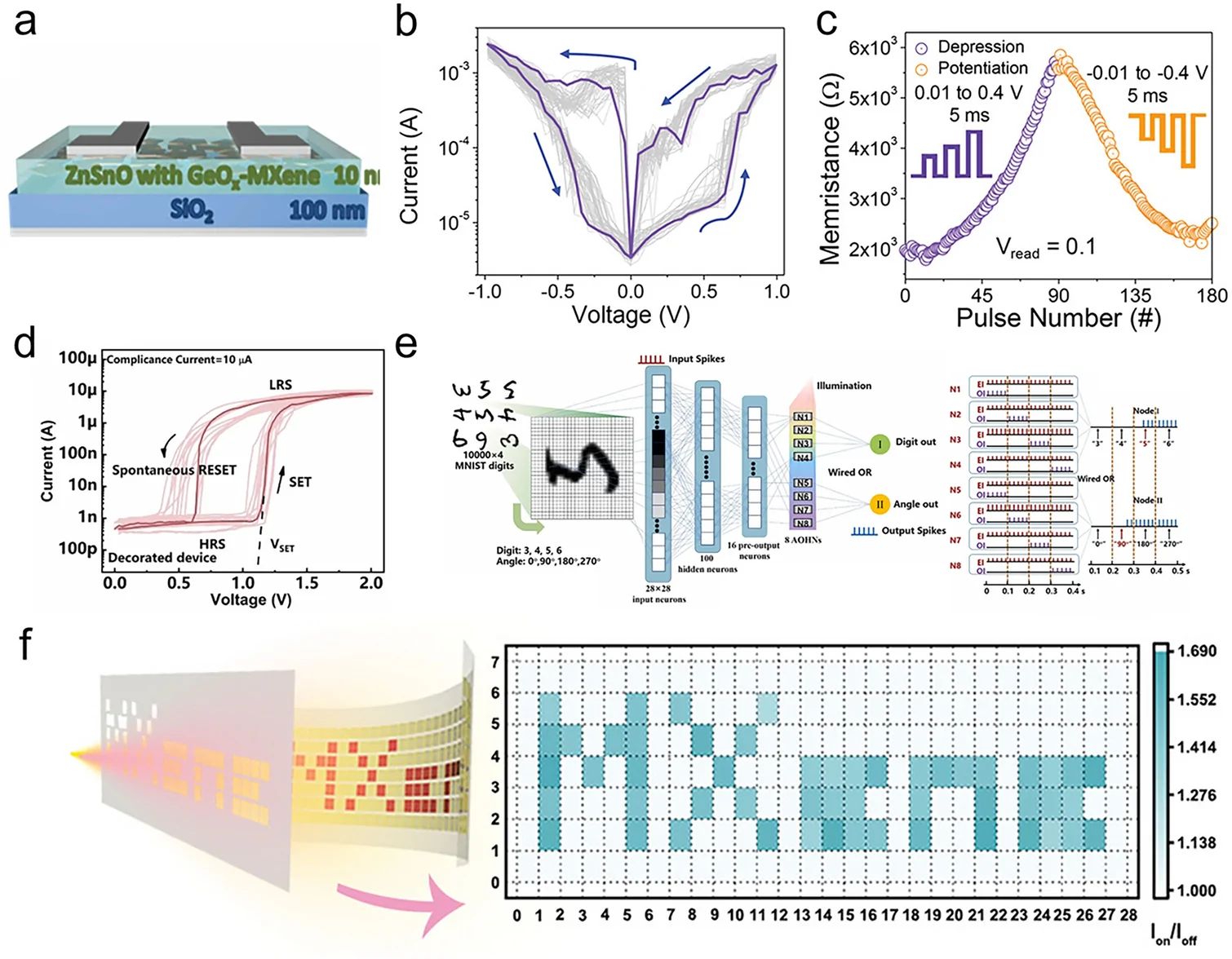
Neuromorphic Visual System Based on MXenes: **a** Schematic diagram of the device structure. Reproduced with permission [103]. Copyright 2023, Elsevier. **b** I–V switching characteristics. **c** Neuromorphic enhancement and inhibition characteristics, as well as spike timing dependent plasticity (STDP) characteristics. Reproduced with permission [100]. Copyright 2023, Wiley‐VCH. **d** I–V characteristic curve exhibiting threshold switching behavior. **e** SNN digital pattern recognition and spatiotemporal integration of EI and OI. Reproduced with permission [101]. Copyright 2022, Elsevier. **f** Example of multi-pixel imaging. Reproduced with permission [102]. Copyright 2023, Wiley‐VCH

The surface-end groups of MXene (such as –O, –F) reduce the potential barrier for electron migration by adjusting the interfacial energy level matching, thereby optimizing the threshold voltage stability of the device. In this process, the movement and accumulation of ions inevitably lead to the formation of charge traps in the active layer, which is one of the reasons for the realization of switchable photovoltaic or memristive behaviors. This effect can be effectively harnessed for data storage and processing. Figure 7a illustrates the structural schematic of the neuromorphic visual device [100]. By applying a small voltage to the device's surface, the switching effect of resistance is excited (Fig. 7b), eliminating the need for inserting electron transport layers and hole transport layers, thereby achieving a stable bipolar resistive switching (RS) loop [100]. To simulate the potentiation and depression learning characteristics of synapses, 90 positive and negative pulses were employed for synaptic function simulation. Figure 7c shows that through synaptic function simulation, MXenes-based optoelectronic devices exhibit plasticity [100]. Furthermore, the broadband light absorption characteristics of MXene and the synergistic effect of the organic photosensitive layer have achieved the dynamic mapping of optical pulse signals and conductance states, providing a multi-physical field coupling platform for visual neuromorphic coding. As depicted in Fig. 7d, under voltage stimulation, the MXenes device demonstrates a lower set voltage and a narrower threshold voltage distribution characteristic [101].

To further validate the capabilities of MXenes in conjunction with neuromorphic characteristics, a spiking neural network (SNN) composed of MXenes sensors, artificial synapses, and neurons was constructed for image recognition (Fig. 7e) [101]. This recognition process involves training by converting image pixels into spike signals, during which neurons receive sequences of electrical spikes from the SNN and are illuminated by ultraviolet light at different time points. Through computation and array processing, light perception can be achieved, successfully producing a 196-pixel (28 × 7) array for imaging the word "MXene" (Fig. 7f) [102]. The potential of MXene sensor arrays and spike-based neural networks in multi-pixel imaging was verified through their combination.

It is worth noting that although the above-mentioned studies have differences in the application scenarios of MXene, their cores all revolve around the tunable electronic density of states (DOS) of MXene and the dynamic regulation ability of surface terminal groups. In future research, the combination of interface engineering and heterogeneous integration strategies may further break through the bottlenecks of response speed and energy efficiency of MXene devices, laying the foundation for the practical deployment of bionic vision systems.

### Neuromorphic Tactile Systems

Multifunctional tactile neurons integrate pressure sensors, memristors, and neuromorphic computing technologies, achieving significant advancements in human–computer interaction systems in recent years. However, traditional independent pressure sensors typically adjust through simple electrical signals, which imposes considerable limitations on their application in perceptual computing [104–107]. Consequently, the integration of artificial synaptic devices based on neuromorphic computing with sensors into intelligent tactile systems within multimodal sensing electronic skin has garnered widespread attention [108, 109].

**Fig. 8:**
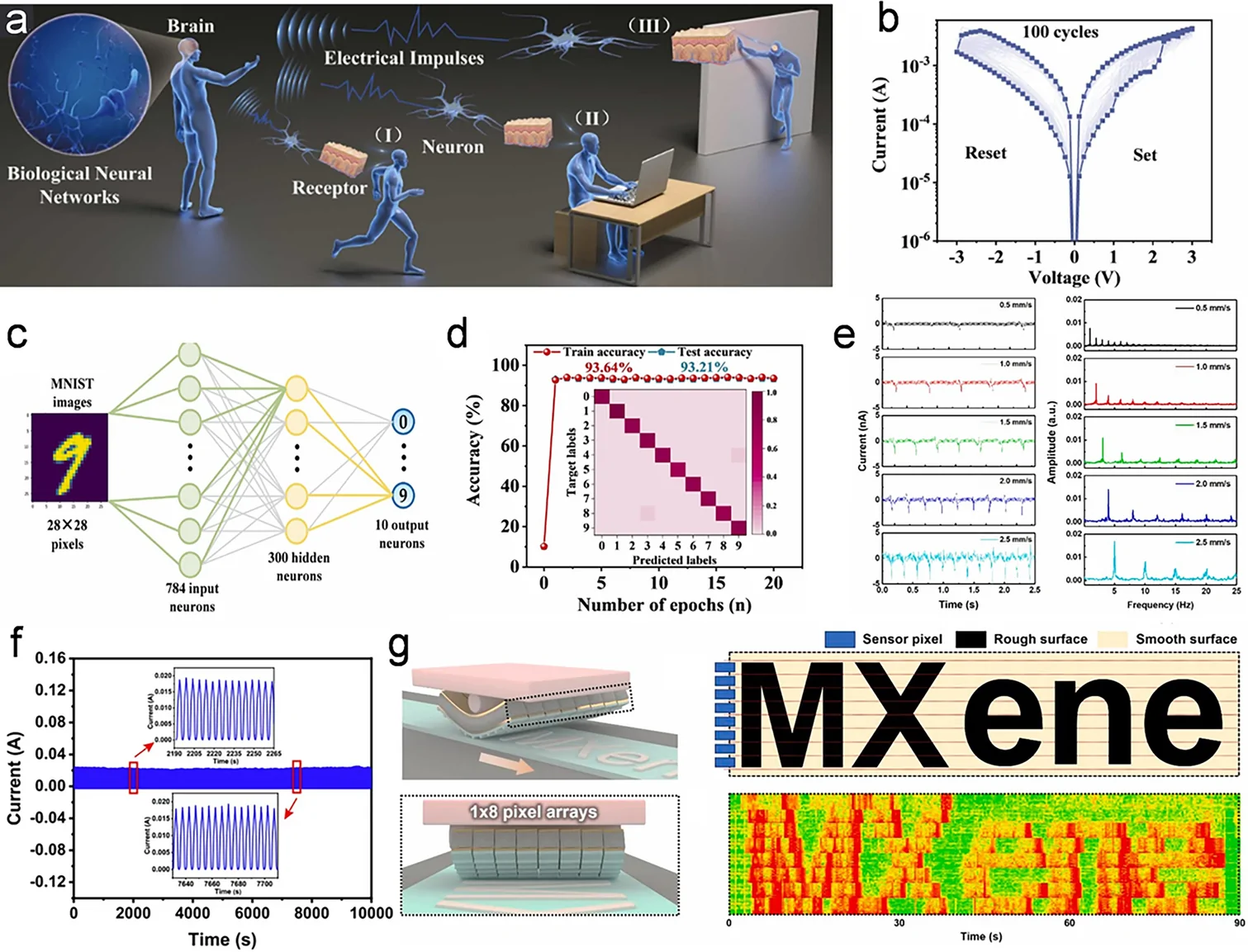
MXenes-based neuromorphic tactile system: **a** Schematic diagram of the biological tactile processing system, where environmental and self-signals are converted into electrical impulses and transmitted to the brain for processing. **b** I-V curve after 100 voltage scans. **c** Schematic diagram of the neural network used for handwritten digit recognition. **d** Recognition accuracy of the training set after 20 epochs. Reproduced with permission [57]. Copyright 2024, Elsevier. **e** Scanning patterned surfaces at speeds of 0.5–2.5 mm s^−1^, observing changes in piezoelectric current and FFT. Reproduced with permission [114]. Copyright 2021, Elsevier. **f** Stability test after 10,000 cycles. Reproduced with permission [115]. Copyright 2023, Springer Nature. **g** Schematic diagram of the MXenes electronic skin array fingerprint patterns. Reproduced with permission [114]. Copyright 2021, Elsevier

In the human body, the system relies on multifunctional neurons to convert external physical stimuli into electrical impulses, which are transmitted to the cerebral cortex via neural networks for further perception, learning, and response [110, 111]. When the brain needs to assess the body's health status, it must receive information from various receptors for comprehensive analysis [110, 112, 113]. By processing and interpreting feedback information, the brain can ascertain the individual's condition (such as exercising, working, or being frail) and respond accordingly (as illustrated in Fig. 8a) [57]. The tactile system based on MXenes materials continues to exhibit stable multi-cycle resistive switching characteristics after 100 bipolar cycling voltage tests (Fig. 8b) [57]. Combining the properties of neuromorphic synapses, an artificial neural network (ANN) was constructed for handwritten digit recognition, achieving an accuracy rate of 93.21% (Fig. 8c, d) [57]. As shown in Fig. 8e, the intensity and quantity of periodic spikes significantly increase with the scanning speed [114].

Moreover, the MXenes tactile system demonstrates exceptional stability, with the current remaining stable after 2000 cycles of testing (Fig. 8f) [115]. As depicted in Fig. 8g, the MXenes smart tactile system can perceive the spatial distribution of different surface textures and their perceptual intensity, distinguishing various roughness levels based on the intensity of piezoelectric current output. Thus, this system enables precise detection of surface features. In Fig. 8g, red and green represent surfaces of high and low roughness, respectively [114].

### Neuromorphic Olfactory Systems

Inspired by biological sensory principles, artificial synaptic electronic devices have demonstrated extensive multisensory functionality within neuromorphic computing platforms [116–121]. While significant advancements have been made in biologically-inspired tactile sensing and visual systems, neuromorphic olfactory systems remain in the exploratory phase [122–125]. The olfactory system plays a crucial role in the perception of environmental information, odor intensity assessment, and even toxicity detection. However, traditional gas sensors typically convert gas signals into electrical signals and require data to be uploaded to the cloud for further identification and analysis, a process that is both complex and inefficient [126, 127]. Moreover, conventional sensors often struggle to accurately distinguish between multiple gases and their concentrations under varying humidity conditions. The calibration of humidity effects is essential for many practical applications beyond just gas detection.

**Fig. 9:**
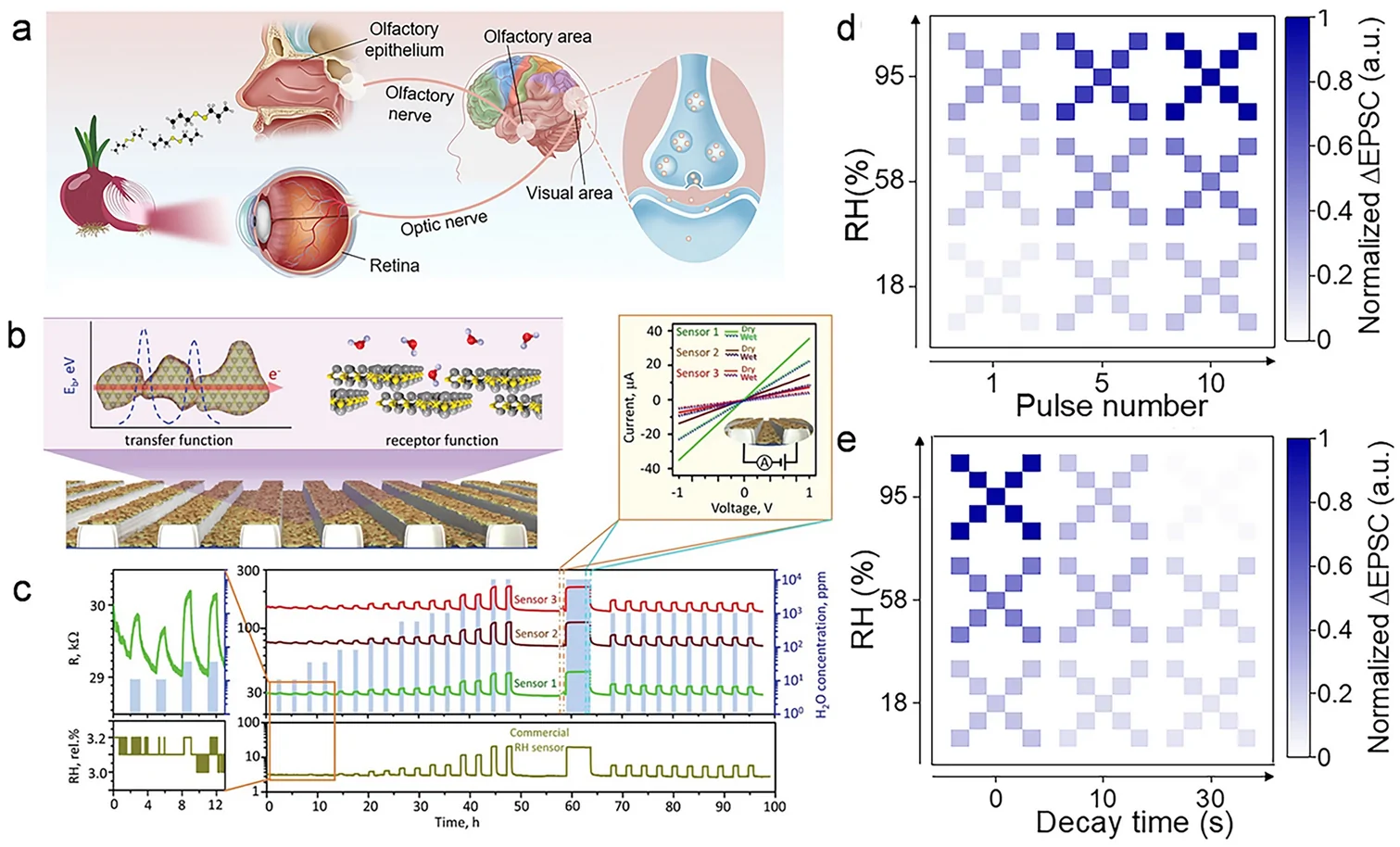
MXenes-based intelligent olfactory system: **a** Schematic diagram illustrating the relationship between vision and olfaction in the recognition of the same object within the human perceptual system. Reproduced with permission [129]. Copyright 2024, Springer Nature. **b** General concept of measuring effects with multi-chip electrodes, indicating that the transfer function is driven by electron transport controlled by potential barriers, with the receptor function dependent on the adsorption process of the analyte on the crystal surface. **c** Measured transient R(t) under multiple exposures to different concentrations of water vapor at room temperature, compared to results from commercial capacitive sensors. The upper right inset shows the I-V curves measured under dry and high humidity conditions. Reproduced with permission [130]. Copyright 2021, Wiley‐VCH. **d**, **e** Analyze the recognition and forgetting processes under different relative humidity (RH) environmental patterns. Reproduced with permission [129]. Copyright 2024, Springer Nature

With ongoing technological advancements, intelligent olfactory systems based on artificial synaptic characteristics and neuromorphic computing can directly convert gas signals into electrical signals for identification, thus circumventing the complex processes of uploading, analyzing, and downloading characteristic of traditional systems [128]. Thanks to the excellent hydrophilicity, large specific surface area of two-dimensional structures, and unique electronic properties of MXene materials, MXene synapses exhibit potential for responding to changes in atmospheric conditions in gas sensing applications. Figure 9a illustrates the interaction between vision and olfaction in the human perceptual system when recognizing the same stimulus. In visual information processing, the activation of specific odors may enhance or suppress visual perception [129]. The chemical resistance effect in MXene layers is caused by electron barrier transport, a mechanism that determines the transfer function of the sensor, as shown in Fig. 9b [130].

By comparing with commercial sensors, Fig. 9c shows the impedance changes of three different MXene sensors under varying H_2_O concentrations, demonstrating good reproducibility and reversibility [130]. A positive correlation is observed between transient resistance and gas concentration. Under high humidity conditions, these sensors maintain stable signal output even after continuous operation for several days. The inset in the upper right corner depicts linear ohmic behavior under two different environmental conditions, ruling out the possibility of large barrier formation at the MXene heterojunction interfaces [128]. The increase in excitatory postsynaptic current (EPSC) is associated with a decrease in dark current, while the shortening of decay time may be attributed to the combination of dissociated H⁺ and OH⁻ from water molecules with electrons and holes, thereby accelerating the disappearance process of captured photogenerated carriers. Figure 9d, e illustrates the recognition and forgetting processes of the letter "X" under different relative humidity (RH) conditions, with results indicating that as RH levels (i.e., odor concentration) increase, both the rate of EPSC rise and decay significantly accelerate [129].

### Comparative Analysis of MXene with Mainstream Memristive Materials

To further emphasize the unique advantages of MXenes in neuromorphic applications, we conducted a comprehensive comparison of MXenes with other mainstream memory materials (such as HfO_*x*_, TaO_*x*_, and MoS_2_) in terms of retention, linearity, switching speed, and multi-level modulation capability (Table 4).

**Table 4:** Performance comparison of MXenes with mainstream memristive materials

Material	Retention	Linearity	Switching speed	Multi-level states	References
Ti_3_C_2_T_*x*_ MXene	> 10^4^	–	100 μs	21-bit	[131]
Ti_2_AlN/HfO_*x*_/Ti	6000	~ 0.3	–	9-bit	[132]
Pt/TaO_*x*_/TiN	> 10^4^	~ 0.2	–	5-bit	[133]
Ge_3_Se_7_	> 10^6^	–	25 ns	6-bit	[134]
MoS_2_	> 10^5^	–	100 μs	–	[135]

Compared with HfO_*x*_ (~ 6000 cycles) and TaO_*x*_ (~ 10^4^ cycles), MXenes exhibited excellent retention capacity (~ 10^4^ cycles), which was attributed to their strong interlayer ion migration kinetics and defect-resistant surface termination. The linearity (nonlinearity factor < 0.3) of memristor conductance modulation based on mxene can provide accurate synaptic weight updates for neuromorphic algorithms. In addition, MXenes achieves an ultrafast switching speed (< 100 μs), comparable to MoS_2_, while maintaining low energy consumption (< 1 pJ/peak) due to its metallic conductivity.

It is worth noting that MXenes performs exceptionally well in multi-level modulation through surface terminal engineering and heterogeneous structure design. For instance, MXene/ polymer composites exhibit a 21-bit analog state, surpassing the 4–6 bit analog states of traditional materials such as Ge_3_Se_7_ and Pt/TaO_x_/TiN. This tunability stems from the tunable electron density of states (DOS) and intercalation-driven ionic dynamics of MXenes, which is not obvious in oxide-based systems.

## Integration of Multimodal Intelligent Systems

Humans acquire environmental information through various sensory modalities such as vision, olfaction, and haptics, as well as their cross-modal interactions, subsequently transmitting this information to the brain for processing to elicit appropriate responses [136–139]. The integration of sensory information enhances environmental perception, facilitating functions such as emotional regulation, scene memory, and learning. These processes rely on the complex multimodal plasticity mechanisms, adaptive computational abilities, and the synergistic effects of evolutionary processes within networks of neurons and synaptic plasticity. Inspired by the human multisensory system, neuromorphic sensing and computing systems combine biomimetic sensors with neural networks to perceive and process various sensory inputs, achieving cross-modal integration [140–143]. However, research at the device level remains insufficient. Therefore, the development of visual-olfactory-haptic cross-modal perceptual optoelectronic synapses capable of simulating multisensory interactions holds significant academic value and promising application prospects for constructing neuromorphic visual systems.

**Fig. 10:**
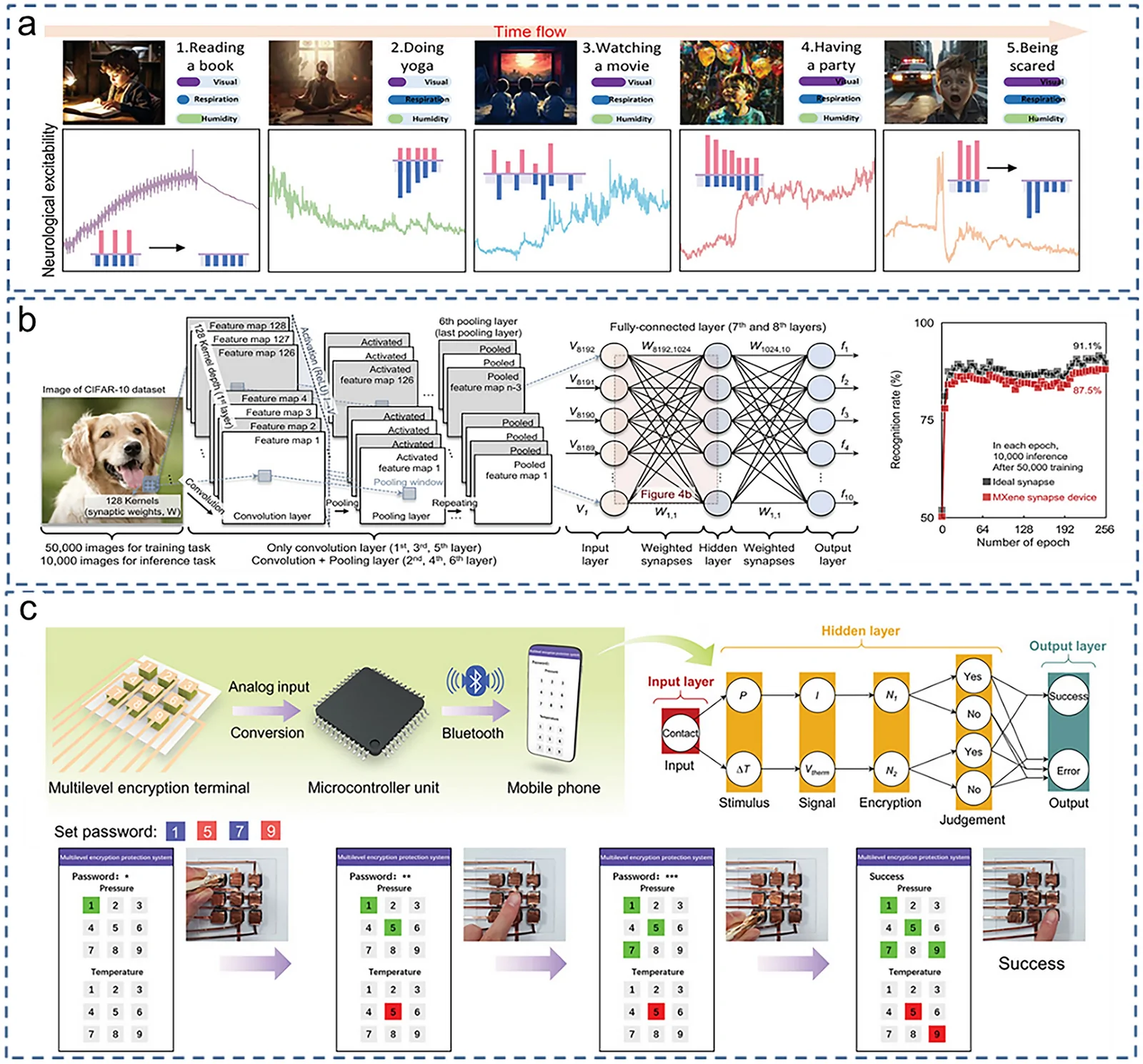
MXenes multimodal intelligent system: **a** Visual and respiratory signal patterns in different scenarios. The PSC triggered by multimodal pulse sequences exhibits varying presentation patterns and integrated excitatory and inhibitory characteristics, replicating neural excitability in different contexts. Reproduced with permission [25]. Copyright 2024, Wiley‐VCH. **b** Convolutional neural network composed of MXenes synaptic devices. Reproduced with permission [144]. Copyright 2021, Wiley‐VCH. **c** Preparation of a multi-layer encryption protection system using a haptic system, including its working mechanism and practical demonstration. Reproduced with permission [145]. Copyright 2024, Wiley‐VCH

Figure 10a illustrates the modulation effects of multimodal signals on neural excitatory signals across five different activity scenarios [25]. These representative scenes correspond to varying intensities of visual input and amplitudes of respiratory relaxation; during relaxation activities (such as reading and yoga), the amplitude of respiration exceeds that of visual input, and the frequencies also differ. Conversely, in states of excitement (such as watching movies, in fearful situations, or attending parties), visual input significantly surpasses the amplitude of respiratory relaxation, with distinct frequency characteristics. Combined with the neural network shown in Fig. 10b, the accuracy of action judgment can reach 91.1% [144]. Furthermore, the integration with intelligent haptic systems enables the development of a multi-level encryption protection system. As depicted in Fig. 10c, the input terminal acquires pressure signals through haptic perception, which are processed and read by a microcontroller, then wirelessly transmitted to a mobile phone using bluetooth technology [145]. This input terminal is simultaneously stimulated by pressure and temperature changes, with these stimuli converted into current and voltage signals, respectively. Subsequently, these electrical signals are transformed into digital data and subjected to encryption. Ultimately, the system can only unlock successfully when both passwords are correct.

**Fig. 11:**
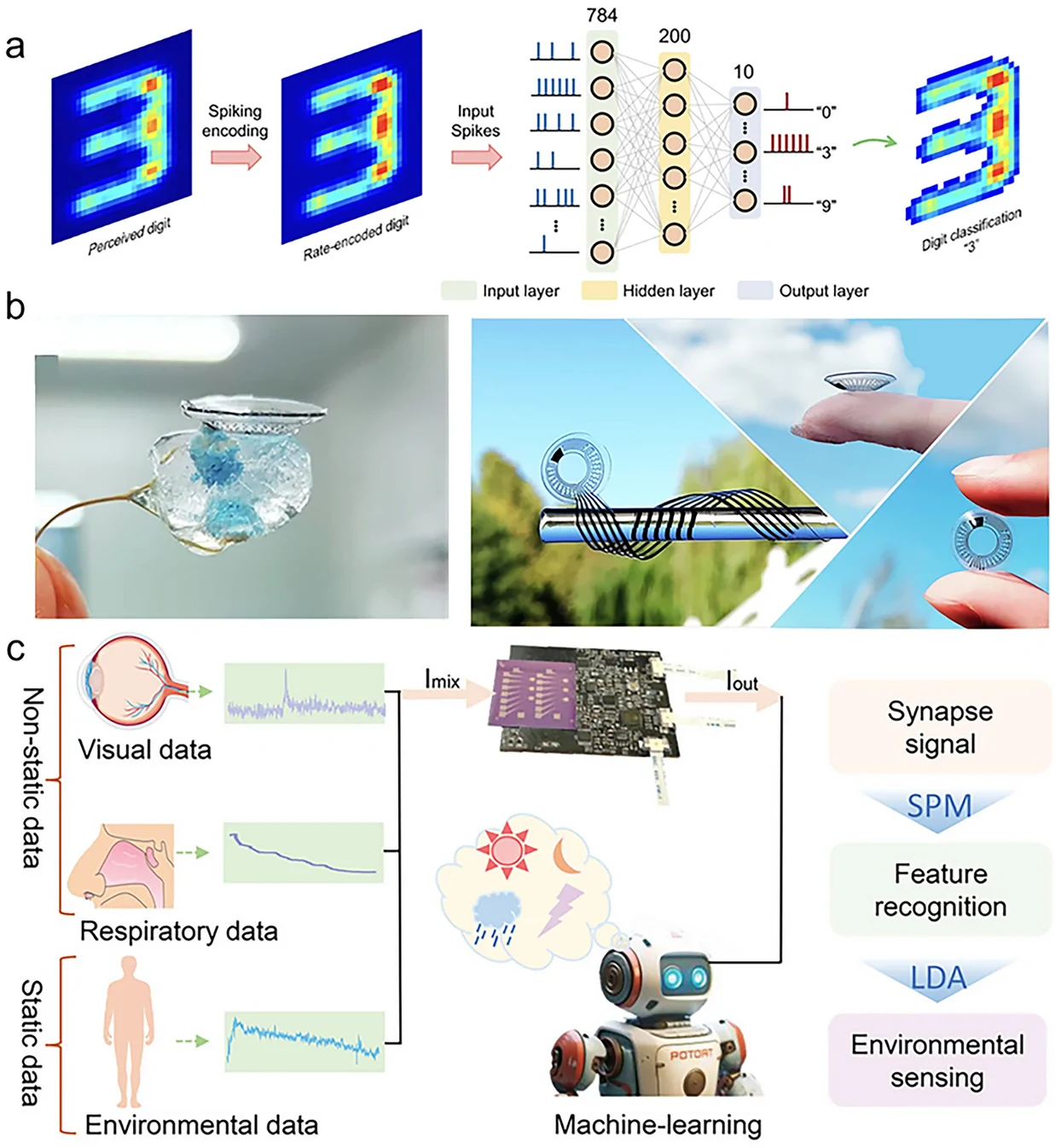
Integration and applications of MXenes multimodal systems. **a** Conversion of detected information into a series of spike sequences, input to a fully connected spiking neural network (SNN) to complete recognition tasks. The number corresponding to the output neuron with the highest spike rate represents the predicted result. Reproduced with permission [148]. Copyright 2023, Springer Nature. **b** Photographs of multimodal intelligent contact lenses for pressure, temperature, etc. Reproduced with permission [149]. Copyright 2024, Springer Nature. **c** Schematic diagram of a biomimetic multi-intelligent system integration for scene recognition and information flow processing, involving multimodal data acquisition processes. Reproduced with permission [25]. Copyright 2024, Wiley‐VCH

Finally, this study integrates MXenes devices with third-generation spiking neural networks (SNNs), which are considered to be more efficient than traditional artificial neural networks (ANNs). This efficiency arises from the significantly lower demand for high-precision multiplication operations in SNNs compared to ANNs. Furthermore, the binary spike density of SNNs is considerably lower than that of ANNs, which helps effectively reduce storage and energy requirements [146]. Relevant research indicates that SNNs operating on spiking neuromorphic hardware can achieve an energy-delay product that is four orders of magnitude lower than that of conventional deep neural networks (DNNs) [147]. As illustrated in Fig. 11a, the fully connected SNN allows the MXenes multimodal intelligent system to transform detected information into a sequence of spikes, thereby completing recognition tasks [148]. The highest spike frequency of the output neurons corresponds to the predicted results. Figure 11b presents an application of integrated multimodal MXenes-based neural prosthetic contact lenses, with the left side showing a photo of the wearer, demonstrating good biocompatibility, and the right side highlighting the aesthetic appeal and transparency of the MXenes device [149]. Through multiple MXenes sensor arrays and data reading circuits, we can implement multimodal sensing circuits based on principles of biological neuromorphology, as shown in Fig. 11c [25]. This system is capable of simultaneously processing dynamic signals from vision and respiration, along with static body data. The output current signals are transmitted to a signal acquisition module for further analysis and sent to a computer for feature extraction. By combining machine learning algorithms with linear discriminant analysis models, we can identify and reproduce complex emotional actions [138, 150]. The neural excitation triggered by light stimulation, the relaxation effects induced by respiration, and the fundamental signal variations caused by body movements enable the interactive devices based on intelligent MXenes technology to simulate human emotions and behaviors. By adjusting light pulses (such as intensity, frequency, duration), airflow pulses (such as hydroxyl content, frequency, and duration), and near-static bodily signals, various combinations of action signals can be generated in specific scenarios.

## Outlook and Challenges

**Fig. 12:**
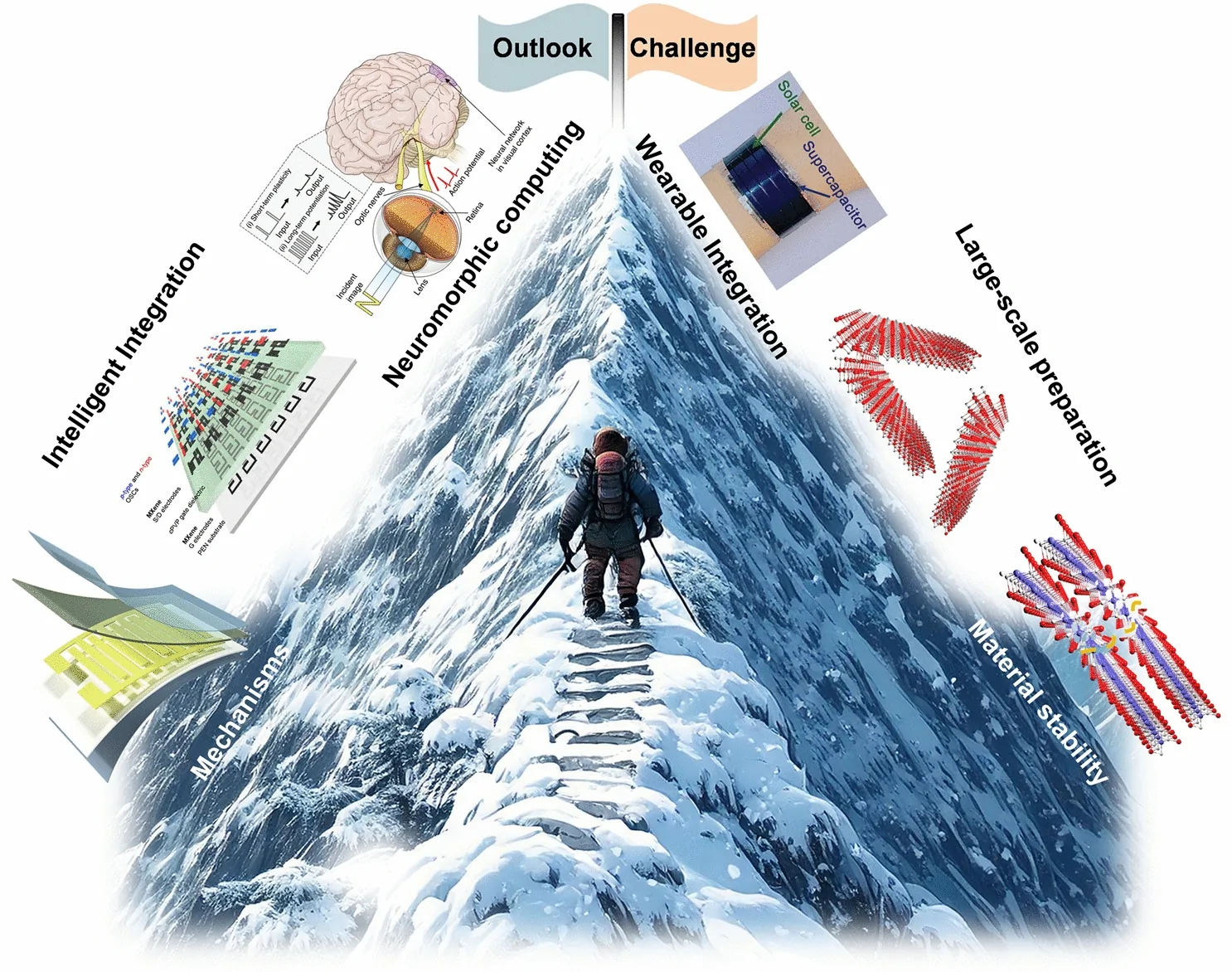
Outlook and challenges of MXenes in smart system integration. MXenes has achieved good development in locations intelligent integration, neuromorphic computing, and mechanisms. Reproduced with permission [151]. Copyright 2020, Springer Nature. Reproduced with permission [152]. Copyright 2019, American Chemical Society. There are still significant challenges at locations wearable integration, large-scale preparation, and material stability. Reproduced with permission [153]. Copyright 2019, Wiley‐VCH

In summary, this study provides a comprehensive review of the current applications of MXenes in the integration of multimodal intelligent systems. As a class of two-dimensional materials with unique physicochemical properties, MXenes exhibit significant application potential in areas, such as optoelectronic detection, gas sensing, and pressure sensing. Despite the ongoing expansion of their applications in the integration of multimodal intelligent systems, particularly the new opportunities arising from their incorporation into neuromorphic computing, the commercialization of MXenes still faces numerous challenges. As shown in Fig. 12, this paper presents the prospects and challenges for the future development of MXenes-based neuromorphic intelligent systems.

### Outlook

1. Intelligent integration: The integration of MXenes in multimodal intelligent systems involves the acquisition and processing of sensory information, including visual, olfactory, and tactile inputs. Thanks to their exceptional sensitivity, rapid response capabilities, and excellent biocompatibility, MXenes offer potential for achieving high-precision, low-power multimodal sensing systems. Meanwhile, the self-powered characteristics of MXene combined with the low power consumption characteristics of neuromorphic devices are expected to promote the development of passive intelligent systems. Future research needs to further explore the potential of MXene in various energy harvesting mechanisms (such as photothermal and thermoelectric), and optimize its compatibility with flexible circuits.
2. Neuromorphic computing: Neuromorphic computing, which aims to mimic biological neural systems, seeks to enhance the energy efficiency and processing capabilities of computers. The application of MXenes in this field primarily focuses on the construction of efficient synaptic elements and neural networks. With their superior conductivity and electrochemical properties, MXenes contribute to the development of high-performance neuronal networks, enabling rapid information processing and complex pattern recognition. Additionally, the low power consumption and good long-term stability of MXenes networks provide strong support for the practical applications of neuromorphic computing.
3. Mechanisms: The operational mechanism of MXenes in the integration of multimodal intelligent systems relies on the synergy of their unique physicochemical properties with neuromorphic computing. Through the cooperative interaction between MXenes and neuromorphic computing, systems can simulate the complex information processing processes of biological neural systems, thereby achieving efficient computational and learning capabilities. MXenes sensors can convert multimodal information from the environment (such as visual, olfactory, and tactile signals) into electrical signals, which are then processed and recognized by MXenes-based neuromorphic computing systems, ultimately producing corresponding outputs to trigger feedback. This mechanism not only enhances the sensitivity and accuracy of the system but also reduces energy consumption and costs.

### Challenges

Despite the significant progress made in integrating MXenes into multimodal intelligent systems, several technical challenges remain that urgently need to be addressed:

1. Wearable integration: The successful incorporation of MXenes into the development of wearable devices is crucial. However, achieving this goal necessitates resolving issues related to the large-scale fabrication and stability of MXenes on flexible substrates. Future research should focus on the flexible processing techniques of MXenes and their integration with flexible electronic devices to promote widespread application in the wearable technology sector.
2. Large-scale preparation: Currently, the production of MXenes primarily relies on chemical etching methods, which are limited by low yield and high costs. Therefore, there is an urgent need to develop new fabrication processes and technologies, such as physical vapor deposition and chemical vapor deposition, for the production of MXene films. Additionally, exploring composite fabrication methods involving MXenes and other materials may enhance their overall performance and broaden their application scope.
3. Material stability: The stability of MXenes directly impacts their performance in practical applications. Environmental factors may lead to degradation or performance decline during long-term use. Thus, it is essential to conduct in-depth research on the stability mechanisms of MXenes and the factors influencing them. Surface modification can enhance their oxidation resistance and corrosion resistance, while exploring composite applications with other materials can further improve their overall stability.

Although MXenes performs well in terms of conductivity and tunable electronic characteristics, its integration with traditional CMOS processes still faces challenges. The thermal stability of MXenes (typically below 400 °C) limits its application in high-temperature annealing processes. Recent studies have successfully achieved the patterned growth of MXene films on silicon-based wafers through low-temperature plasma-enhanced chemical vapor deposition (PE-CVD) technology, providing a new idea for hybrid integration. In addition, the interface contact resistance between MXene and metal electrodes (such as Au, TiN) needs to be further optimized to match the performance requirements of standard logic devices. The mechanical compatibility of MXenes with flexible polymer substrates such as PDMS and PET is of crucial importance. For packaging issues, Al₂O₃ or HfO₂ films deposited by atomic layer deposition (ALD) can effectively prevent the penetration of water and oxygen, extending the environmental stability of MXene devices to more than six months. At present, the large-scale preparation of MXene mainly relies on the wet chemical etching method, but this process has problems such as complex waste liquid treatment and low yield. Emerging gas-phase exfoliation techniques (such as the molten salt-assisted method) can increase the yield of monolayer MXene to 80% and are suitable for roll-to-roll continuous production. In the future, it is necessary to further develop green synthetic routes, reduce production costs, and promote the transformation of MXene from the laboratory to industrial applications.

Overall, MXenes exhibit tremendous potential and a wide range of application prospects in the field of multimodal intelligent system integration. However, a series of critical technical issues must be resolved to facilitate the widespread application and commercialization of MXenes. Future research will focus on flexible processing technologies, large-scale fabrication methods, and long-term stability, thereby advancing their applications in intelligent integration and neuromorphic computing.

Looking ahead, further exploration of composite usage technologies involving MXenes and novel materials may lead to the development of higher-performance, broader application multimodal intelligent systems. With the continuous advancement of artificial intelligence and Internet of Things technologies, MXene-based multimodal intelligent systems will deeply integrate with cutting-edge technologies, jointly driving innovation and development in the fields of intelligent perception and interaction. It is believed that, in the near future, MXenes will play an increasingly important role in the integration of multimodal intelligent systems and contribute more significantly to social progress.
